# Regulation of lineage reprogramming by dynamic chromatin SUMOylation

**DOI:** 10.1007/s00018-026-06255-5

**Published:** 2026-05-27

**Authors:** Emma Valima, A. B. M. Kaiser Manjur, Eevi Savinainen, Vera Varis, Kaisa-Mari Launonen, Thomas Graf, Markku Varjosalo, Einari A. Niskanen, Jorma J. Palvimo

**Affiliations:** 1 Institute of Biomedicine, School of Medicine, University of Eastern Finland, Kuopio, Finland; 2 Centre for Genomic Regulation, The Barcelona Institute of Science and Technology, Barcelona, Spain; 3 Institute of Biotechnology, HiLIFE Helsinki Institute of Life Science, University of Helsinki, Helsinki, Finland

**Keywords:** B-cell, CEBPA, BLaER1, Macrophage, ML-792, Transdifferentiation

## Abstract

SUMOylation has emerged as a key regulator of chromatin and transcription, yet its contribution to lineage reprogramming remains unclear. To explore how chromatin SUMOylation influences cellular plasticity, we studied CEBPA-driven lineage reprogramming of human leukemic B-cells into macrophage-like cells. By integrating ChIP-seq, ATAC-seq, RNA-seq and chromatin-directed proteomics, we mapped the chromatin landscape and transcriptomic changes during early reprogramming. Lineage conversion triggered a dynamic rise in SUMO2/3 chromatin occupancy at CEBPA-bound sites, revealing a coordinated regulatory mechanism. Proteomic profiling of SUMO2/3- and CEBPA-associated chromatin uncovered extensive convergence and enrichment of differentiation-related transcription factors, chromatin remodelers and coregulators. Among these, NCOA3 displayed markedly increased SUMO2/3 association upon lineage conversion. NCOA3 co-occupied CEBPA- and SUMO2/3-bound chromatin regions, implying a SUMOylation-supported coregulatory role in lineage reprogramming. Pharmacological inhibition of SUMOylation using ML-792 (SUMOi) selectively enhanced CEBPA chromatin occupancy and chromatin accessibility, altered the CEBPA association of proteins, and modified NCOA3 binding dynamics. SUMOi also reshaped gene expression, promoting loss of B-cell identity and activation of macrophage-associated programs, including lipid metabolism. Collectively, our findings highlight chromatin SUMOylation as a dynamic and context-dependent modifier that fine-tunes lineage transitions, with implications for chromatin biology and therapeutic modulation of cell identity.

**Supplementary Information:**

The online version contains supplementary material available at 10.1007/s00018-026-06255-5.

## Introduction

The tightly orchestrated interplay between transcription factors (TFs), coregulators, chromatin remodeling factors and chromatin states governs gene programs determining cell differentiation and fate [1]. Reversible post-translational modifications (PTMs) provide an additional regulatory layer by modulating protein–protein interactions (PPIs) and chromatin functions. Although cell differentiation and cell fate transitions involve extensive reorganization of gene expression, the role of chromatin-associated PTMs beyond histone modifications in these processes remains poorly understood. Notably, SUMOylation has emerged as a general stabilizer of cell identity, counteracting the reprogramming of mouse embryonic fibroblasts (MEFs) into induced pluripotent stem cells (iPSCs) and maintaining both somatic and pluripotent states [2–4].

SUMOylation, the covalent attachment of small ubiquitin-like modifier (SUMO) proteins to target lysines in substrates is a dynamic and essential PTM that regulates a wide array of cellular processes, particularly nuclear ones, such as transcription and genomic integrity [5–7]. The SUMO family comprises three major isoforms, SUMO1, SUMO2, and SUMO3, with SUMO2 being the predominant and essential isoform nearly identical to SUMO3 (hence they are collectively referred to as SUMO2/3) [8, 9]. SUMO conjugation proceeds via a dedicated enzymatic cascade involving the E1-activating enzyme (SAE1/2), the E2-conjugating enzyme (UBC9), and multiple E3 ligases, such as PIAS1–4, and is reversed by SUMO-specific proteases (SENPs) [6, 10, 11].

TFs and chromatin-associated proteins constitute major classes of SUMOylation targets, underscoring the modification’s role in gene regulation [6, 12]. Chromatin-bound SUMO landscapes differ markedly between MEFs and iPSCs and during adipogenesis [3, 13], and are dynamically reshaped by environmental cues such as heat shock, steroid hormones, and inflammatory signals. These SUMO2/3 redistribution events correlate with changes in transcriptional programs, linking SUMOylation to cell-state-specific gene regulation [14–16].

Despite these insights, the chromatin-wide mechanisms by which SUMOylation influences transcriptional reprogramming and cell fate transitions remain incompletely understood. We hypothesized that during cell fate conversions initiated by master TFs, SUMOylation regulates chromatin-bound protein networks surrounding these factors, thereby influencing the rewiring of their transcriptional programs. To address this, we investigated the role of chromatin SUMOylation in a CCAAT/enhancer-binding protein alpha (CEBPA)-inducible B-cell-to-macrophage transdifferentiation system, a well-established model of lineage reprogramming [17].

By integrating chromatin binding, accessibility and proteomics, and transcriptomic analyses with pharmacological SUMOylation inhibition, we show that dynamic SUMO2/3 modification acts as a temporal regulator of the CEBPA chromatin network and transcriptional programs, restraining premature macrophage gene activation and shaping the trajectory of the cell fate transition.

## Materials and methods

### Cell culture

BLaER1 cells (pre-B-cells containing a fusion of CEBPA with the estrogen receptor (ER) hormone binding domain) [17] were maintained in RPMI-1640 medium supplemented with 25 mM HEPES, 10% (v/v) heat-inactivated fetal bovine serum, 1 U/µl penicillin, 1 µg/ml streptomycin, 2 mM L-glutamine and 0.1% (v/v) 2-mercaptoethanol (all from Gibco, Waltham, MA, USA). Cells were routinely tested for mycoplasma contamination.

### B-cell-to-macrophage transdifferentiation

BLaER1 cells were seeded at a density of 5 × 10^5^ cells/ml in maintenance medium. After 24 h, transdifferentiation was induced by treating the cells with 100 nM β-estradiol (E2) (Calbiochem, San Diego, CA, USA) in the presence of 10 ng/ml of hrIL-3 and 10 ng/ml hrCSF-1 (PeproTech, Cranbury, NJ, USA). In SUMOylation inhibition studies, cells were exposed to 100 nM ML-792 (SUMOi; 407886, MedKoo Biosciences Inc., Morrisville, NC, USA) or an equivalent volume of DMSO (vehicle control) for 24 h prior to induction.

### Antibodies

Used antibodies were anti-CEBPA (purified rabbit IgG produced in-house in Graf lab), anti-SUMO2/3 (M114-3, MBL International Corporation, Woburn, MA, USA), anti-NCOA3 (A300-347A, Bethyl Laboratories), normal rabbit IgG (12–370, Merck Millipore, Burlington, MA, USA), normal mouse IgG (sc-2025, Santa Cruz Biotechnology, Santa Cruz, CA, USA), and anti-lamin B (ab16048, Abcam, Cambridge, United Kingdom).

### Immunoblotting

Prior to lysate preparation, cells were counted using a Bio-Rad automated cell counter. Cells were harvested by centrifugation and cell pellets were washed twice with 1 ml of phosphate-buffered saline containing 1 × protease inhibitor cocktail (PIC, Roche, Basel, Switzerland) and 10 mM N-ethylmaleimide (NEM). The washed cells were pelleted by centrifugation at 16 000 × g for 30 s at 4 °C. Cell pellets were resuspended in 1 × SDS sample buffer supplemented with 1 × PIC, 10 mM NEM, and 5% (v/v) 2-mercaptoethanol. A volume of 100 µl of lysis buffer was used per 3 × 10^6^ cells. The lysates were heated at 95 °C for 10 min and vortexed thoroughly to ensure proper solubilization. Samples were sonicated using a microsonicator for two cycles of 10 s each (cycle 1, amplitude 100%). Samples were re-heated for 5 min at 95 °C prior to separation on 10% SDS-PAGE gels, and subsequently proteins were transferred onto nitrocellulose membranes and visualized by antibodies against SUMO2/3 or lamin B. Appropriate horseradish peroxidase-conjugated secondary antibodies (Life Technologies, Carlsbad, CA, USA) and chemiluminescence reagent (Pierce, Waltham, MA, USA) were used for detection.

### MTS assays

BLaER1 cells were plated into 96-well plates at a density of 3 × 10^4^ cells/well, in 5 replicates, and treated with indicated concentrations of ML-792 in a total volume of 100 µl/well. MTS assay was performed using CellTiter 96 AQueous One Solution Cell Proliferation Assay (G3580, Promega, Madison, WI, USA) as described in [18], with 20 µl CellTiter reagent per well.

### ChIP-seq

Chromatin immunoprecipitation (ChIP) was performed as described [19]. Cells at 0 (uninduced), 6 and 24 h after induction of transdifferentiation were fixed for 10 min at room temperature in maintenance medium with 1% (v/v) formaldehyde and then quenched with 125 mM glycine for 10 min. Chromatin was fragmented to an average size of 300–500 bp by sonication (Bioruptor, UCD-300, Diagenode, Liege, Belgium). Antibodies were coupled to magnetic protein G beads (Dynabeads, Invitrogen, Waltham, MA, USA) for 16 h, sonicated lysates were incubated with antibody-coupled beads for 16 h, washed, eluted and de-crosslinked in the presence of proteinase K (Fermentas, Waltham, MA, USA). Antibodies used per immunoprecipitation reaction: SUMO2/3, 2 µg; SRC3, 2 µg; CEBPA 3 µg. ChIP-seq sequencing libraries were generated according to manufacturer’s protocol using NEBNext Ultra II DNA Library Prep Kit (E7645L, New England Biolabs). In general, two biological replicate samples were sequenced at the EMBL Genomics Core Facility (Heidelberg, Germany) using Illumina HiSeq 2000 (50SE).

### ATAC-seq

BLaER1 cells were seeded in 6-well plates 1 × 10^6^ cells/well and treated as for ChIP-seq above. Nuclei isolation was performed as in [18], based on the protocol reported in [19]. The following modifications were made: the pellet was resuspended in ice-cold Buffer A to a final concentration of 5 × 10^6^ cells/ml. To isolate nuclei, an equal volume of Buffer A supplemented with 0.04% (v/v) IGEPAL CA-630 was added, to achieve a final concentration of 2.5 × 10^6^ cells/ml and 0.02% (v/v) IGEPAL CA-630. After incubation on ice for 10 min, the nuclei were pelleted, washed once with ice-cold Buffer A and once with ATAC-Resuspension Buffer (10 mM NaCl, 10 mM Tris–HCl, pH 7.4, 3 mM MgCl_2_). Transposition reaction and PCR amplification were carried out as described in [18]. Immediately after the transposition step, the DNA was purified using the Monarch PCR & DNA Cleanup Kit (T1030, New England Biolabs). Primer sequences are available in [20]. The number of additional cycles needed for each sample was calculated as in [20] by determining the cycle number that corresponded to 1/3 of the maximum fluorescent intensity. Size selection with SPRIselect beads (Beckman Coulter, Brea, CA, USA) was performed according to the manufacturer’s instructions to remove fragments < 150 bp and > 800 bp. Quality of the purified libraries was assessed using a Fragment Analyzer HS NGS kit (474–0500, Agilent, Santa Clara, CA, USA) according to the kit instructions. Prior to sequencing, the purified DNA was stored in DNA LoBind Tubes (Eppendorf, Hamburg, Germany). Two biological replicates were sequenced at the EMBL Genomics Core Facility using Illumina NextSeq 2000 (50PE).

### ChIP-seq and ATAC-seq data analysis

ATAC-seq read filtering was performed as previously described [19, 21]. Subsequently, alignment of paired-end samples to hg38 genome using Bowtie2 and downstream data analysis with HOMER [22] was performed as described in [18]. Briefly, peaks in each dataset were called using findPeaks with style factor, FDR < 0.01, > 25 tags, > 6-fold over local background. ChIP-seq data analysis was performed as previously described [18, 19, 21] and downstream data analysis was performed using HOMER [22]. Tag directories were generated using makeTagDirectory with option -fragLength 200 (for SUMO2/3) and -fragLength 300 (for CEBPA and NCOA3). Peaks in each dataset were called using findPeaks with style factor, FDR < 0.001, > 25 tags, > 4-fold over control sample and local background. Combined tag directory from all ChIP input samples was used as control sample. getDifferentialPeaks was used to isolate differential binding peaks (Poisson *P*-value < 0.0001, FC > 3) between different treatment conditions. Heatmaps were generated with 20 bp bins surrounding ± 1 kb area around the center of the peak. All plots were normalized to 10 million mapped reads and further to local tag density, tags per bp per site. Box plots represent log_2_ tag counts. SUMO2/3 binding sites in DMSO were categorized into shared and overlapping binding sites between analysed timepoints (clusters [C]1–7) using mergePeaks command; CEBPA binding sites in DMSO were categorized similarly into C8–13. CEBPA binding sites in response to SUMOi were categorized into clusters C14–19 based on FC, with C14 and C17 representing binding sites where the difference between DMSO and SUMOi combination was < 3-fold, and C15 and C18 representing those with > 3-fold difference. SUMO2/3 signal upon SUMOi in C17-19 is representative of one replicate. De novo motif searches were performed using findMotifsGenome.pl with the following parameters: 200 bp peak size window, strings with 2 mismatches, binomial distribution to score motif p-values, and 50 000 background regions. Statistical significance was determined with One-way ANOVA with Bonferroni post hoc test. Correlation matrices were generated with deepTools v.3.5.6 [23]. Average scores for ChIP-seq and ATAC-seq datasets were computed using multiBigwigSummary in either bins mode for calculation across the genome in consecutive 10 kb bins or BED-file mode for calculation in C1-7 and C8-13 peak populations. Spearman correlation coefficients were subsequently computed and hierarchically clustered heatmaps generated using plotCorrelation with option -c spearman.

### RNA-seq

Protocol was modified from [24]. BLaER1 cells were harvested at 0 (uninduced), 6, 24, 36, 48 and 72 h after induction. Total RNA was extracted with Monarch Total RNA Miniprep kit (T2010S, New England Biolabs, Ipswich, MA, USA), and mRNA isolated using NEBNext Poly(A) mRNA Magnetic Isolation Module (E7490, New England Biolabs) according to manufacturer’s recommendations. RNA-seq libraries were prepared from three biological replicates using NEBNext Ultra II kit (E7765, New England Biolabs) and pooled libraries were sequenced with HiSeq 2000 at The EMBL Genomics Core Facility.

### RNA-seq data analysis

The sequencing data were processed using an in-house pipeline. Trimmed raw reads were aligned to human genome assembly GRCh38 (hg38) from Genome Reference Consortium using STAR [25]. Reads in exons were quantified using HOMER [22] analyzeRepeats.pl with style rna. Data were collapsed to protein-coding transcripts with the highest read count. Differential gene expression and downstream analysis were performed with TimeSeries Analysis (TiSA) pipeline [26]. Data normalization and differential gene expression were performed with DESeq2, adj. *p*-value < 0.01 and |log_2_FC|> 1.5. Parameters for Partitioning Algorithm based on Recursive Thresholding (PART)-clustering were |log_2_FC|> 1.5, minimum cluster size of 50 and number of recursions 100. For reproducibility, PART seed was set to 30106. Differentially expressed gene (DEG) sets were subjected to pathway analysis with Metascape [27]. Data were visualized R-studio version 2023.06.1 and R v4.4.2, and Adobe Illustrator 2023 (v 28.0).

### Chromatin-directed proteomics (RIME)

Rapid immunoprecipitation mass spectrometry of endogenous proteins (RIME) [28] was performed as described in [18] with minor modifications. BLaER1 cells were seeded into 15-cm culture dishes at 15 × 10^6^ cells per dish in maintenance medium as three biological replicates and then pre-treated and induced as described above and harvested at 0, 6 and 24 h after induction. Crosslinking and cell lysate preparation were performed as in ChIP-seq experiments described above. Fragmented chromatin was incubated with antibody overnight (16 h) at 4 °C, in a total volume of 1 ml RIME-RIPA buffer (prepared in LC–MS grade H_2_O). Antibodies used per chromatin-antibody coupling: anti-SUMO2/3, 1 µg; anti-CEBPA, 3 µg; normal rabbit IgG, 2 µg; normal mouse IgG, 1 µg. Antibody-coupled chromatin was collected on beads in 1.5 ml Protein LoBind tubes (Eppendorf) over 16 h at 4 °C. Enzymatic digestion, peptide de-salting by solid-phase extraction and LC–MS/MS were performed as described in the original RIME protocol [28]. Briefly, beads were washed with ammonium bicarbonate and sequencing grade modified trypsin was added to beads for 15 min with frequent vortexing followed by digestion overnight at 37 °C without further agitation. Subsequent digestion with added trypsin was carried out for 4 h. Peptide digests in supernatant were quenched with trifluoroacetic acid and finally desalted with C18 MicroSpin columns as described in [29]. The dried peptides were reconstituted in Buffer A, diluted 1:20 HPLC water containing formic acid and loaded onto Evotips (Evosep, Odense, Denmark) by manufacturer’s instructions. LC–MS analysis was performed by using the Evosep One liquid chromatography system coupled to a hybrid trapped ion mobility quadrupole TOF mass spectrometer (Bruker timsTOF Pro) via a CaptiveSpray nano-electrospray ion source as described in [18]. The MS analysis was performed in the positive-ion mode using data-dependent acquisition (DDA) in PASEF [30] mode with DDA-PASEF-short_gradient_0.5 s-cycletime -method.

### RIME mass spectrometry analysis

Raw data were processed as described in [18]. FragPipe v17.1 with MSFragger [31] was used to search against reviewed human entries of the UniProtKB database (downloaded 8.3.2022). Results from these steps are spectral counts (SPC) values from peptides with FDR < 0.01 from Philosopher [32]. Protein-matched data were analyzed as in [16]. Probabilistic scoring was performed with SAINT [33], using IgG samples as background controls. Proteins with FDR < 0.05 in DMSO in any timepoint were considered significant; for DMSO vs SUMOi comparisons (CEBPA chromatomes), protein had to have FDR < 0.05 in either DMSO or SUMOi treatment in the compared timepoint. For comparisons between different treatments, SPCs of chromatome members were normalized by bait SPC. Proteins in the SUMO2/3 chromatome with |log_2_FC|> 1 were considered differentially SUMO2/3-associated, and proteins in the CEBPA chromatome with |log_2_FC|> 0.3 were considered differentially CEBPA-associated. STRING database was used to create physical networks and retrieve confidence of interaction scores. Illustrations were prepared with R-studio version 2023.06.1 and R v4.4.2 with ggplot2, Cytoscape (v. 3.10.2) and Adobe Illustrator 2023 (v. 28.0).

### Protein aggregation capture (PAC)

To 20 µg protein equivalents of crosslinked and fragmented RIME input samples (see above) in 10 µl RIPA buffer, an equal volume of lysis buffer (20 mM Tris(2-carboxyethyl)phosphine hydrochloride [TCEP] and 40 mM 2-chloroacetamide [CAA] in 50 mM TEAB) were added to achieve final concentrations of 10 mM TCEP and 20 mM CAA. The samples were subsequently incubated for 1 h at 37 °C, 300 rpm, and centrifuged at 16 000 × g for 10 min. For protein aggregation capture (PAC) [34] purification, 5 µl of MagReSyn Hydroxyl magnetic beads (MR-HYX005, ReSyn Biosciences, Gauteng, South Africa) was added to the supernatant. Acetonitrile was added to a final concentration of approx. 70%, and samples were incubated for 10 min at 22 °C without agitation. After carefully mixing the samples with a pipette, the supernatant was removed. The beads were washed three times with 1 ml of 95% acetonitrile. For on-bead digestion, 100 µl of 100 mM Tris–HCl, pH 8 containing 500 ng of trypsin/Lysc (V5073, Promega) was added to the beads, after which the samples were incubated for 16 h at 37 °C, 500 rpm. Samples were acidified with 10% trifluoroacetic acid (10723857, Thermo Fisher Scientific, Waltham, MA, USA). Peptide concentration was measured using Quantitative Peptide Assays (23275, Thermo Fisher Scientific) and approx. 200 ng was loaded into Evotips (Evosep, Odense, Denmark) according to manufacturer’s instructions. LC–MS/MS analysis was performed by using Orbitrap Astral MS (ThermoFisher Scientific) controlled with Thermo Tune software (v. 1.1.477.46) coupled to an Evosep One (Evosep Biosystems) liquid chromatogram. Samples were analyzed with the 60 SPD method using an 8 cm × 150 µm 4th Generation Aurora Rapid analytical column (IonOptics) interfaced online using an EASY-Spray source. The column oven was set to 50 °C. Mobile phases A and B were 0.1% formic acid in water and 0.1% formic acid in acetonitrile. The Orbtrap Astral MS was operated using the “DominicDIA_60SPD_240k_10ms_3Th_4ms_ 150–2000” method at a full MS resolution of 240 000 with a full scan range of 380–980 m/z. RF lens was set to 45%, and the full-MS AGC target was set to 500%. Maximum injection time for full-MS was 4 ms. MS/MS scans for DIA analysis were recorded with 3 Th isolation window from 380 to 980 m/z, 10 ms maximum ion injection time and AGC target 500%. The isolated ions were fragmented using HCD with 27% normalized collision energy.

### PAC mass spectrometry analysis

Raw Orbitrap Astral DIA data were processed with DIA-NN version 2.3.0 [35]. A spectral library was generated from a human proteome FASTA file (downloaded on 14/05/2025 from UniProtKB/Swiss-Prot database, containing 20,383 proteins), using the following precursor ion generation parameters: Trypsin/P (protease), 1 missed cleavage, cysteine carbamidomethylation enabled as a fixed Modification, and N-terminal methionine excision was allowed as variable Modification, peptide length range from 7 to 30, precursor charge from 1 to 4, 380–980 m/z precursor range, and 120–1500 m/z fragment ion range. For the raw file searches, heuristic protein inference was enabled, and proteotypicity was set to protein names from FASTA. Mass accuracy was set to 10 and for 4 for MS1. Unrelated runs option was not selected, while match-between-runs were selected, and IDs, RT, and IM profiling was chosen for the library generation. Machine learning, quantification strategy, and cross-run normalization were set to cross-validated NNs, QuantUMS, and RT-dependent. FDR for the output filtering was set to 1%. Data output from Dia-NN was further processed with ProteoGyver v.1.5.5 [36]. Proteins not detected in at least 60% of samples in at least one sample group were filtered out. Missing values were imputed with QRILC. Proteins with FDR (Benjamini-Hochberg) < 0.05 were considered significant. For significance testing, maxLFQ |log_2_FC|> 1 was used together with adjusted *p*-value (FDR) threshold of 0.05. Illustrations were prepared with R-studio version 2023.06.1 and R v4.4.2 with ggplot2 and Adobe Illustrator 2023 (v. 28.0).

### Chromatin binding and gene expression integration analyses

SUMOi-altered CEBPA binding site clusters and SUMO2/3 peaks at 0, 6 and 24 h after induction were merged with HOMER [22] mergePeaks command using literal overlap for merging option (-d given). This overlapped two peaks if they are on the same chromosome and their intervals intersect (the start of one peak is less than or equal to the end of the other and the end of one peak is greater than or equal to the start of the other). The overlapping peak populations were then applied to Binding and Expression Target Analysis (BETA) [37] in Cistrome environment. Peaks were integrated into transcriptional alterations at 36 h E2 induction timepoint between DMSO and SUMOi. In BETA analysis, the default parameters were used, except for Differential expressed gene FDR Threshold, which was set to 0.01 and the genome was set as hg38. CTCF boundaries were not considered in filtering the peaks around genes. Pathway analysis was executed for temporal and conditional DEGs (adj. *p*-value < 0.01 & |FC|> 1.5 in Metascape [27] among the predicted target genes in BETA analysis. Association information was complemented to pathway analyzes in Cytoscape.

### Statistical analysis

Statistical significance was determined with GraphPad Prism software unless otherwise stated using the appropriate tests specified above and in each figure legend. The data meet assumptions of population distribution. Variance between the groups that are being statistically compared is similar. Differences were considered significant at *p* < 0.05. Data are presented as mean ± standard deviation. Statistical significance is indicated in the figures as follows: * = *p* < 0.05, ** = *p* < 0.01, *** = *p* < 0.001.

## Results

### CEBPA-directed B-cell lineage conversion triggers dynamic chromatin SUMOylation

**Fig. 1:**
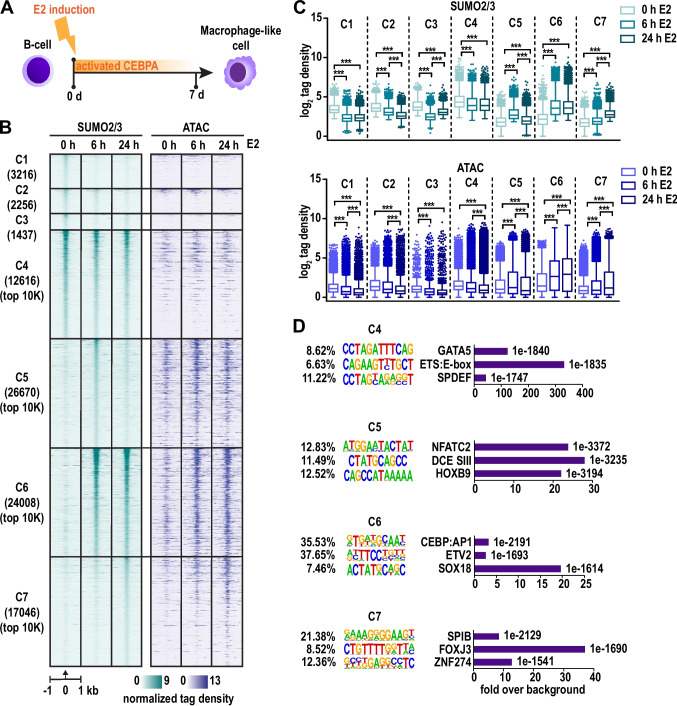
Lineage conversion triggers dynamic chromatin SUMOylation. **A** E2-induced CEBPA drives transdifferentiation from precursor leukemia B-cells to macrophage-like cells. **B** Distinct chromatin regions become SUMOylated during early stages of transdifferentiation. Heatmaps depict SUMO2/3 occupancy on chromatin and chromatin accessibility at indicated timepoints after E2-induction. C1: SUMO2/3-binding sites unique to 0 h; C2: shared between 0 and 6 h; C3: shared between 0 and 24 h; C4: shared between 0–24 h; C5: unique to 6 h; C6: shared between 6 and 24 h; C7: unique to 24 h. For C4-7 sites, only top 10 K are shown. **C** Boxplots of SUMO2/3 and ATAC signal (tag density) in the SUMO2/3 clusters C1-7 depicted in **B**. **D** Top three de novo motifs for C4–C7, showing enrichment as fold over background and respective *p*-values at the end of the bars. Percentages indicate the percentage of SUMO2/3-binding sites with motif. Statistical significance for boxplots was calculated with One-way ANOVA with Bonferroni post hoc test, with asterisks denoting statistical significance: * = *p* < 0.05, ** = *p* < 0.01, *** = *p* < 0.001

To investigate the dynamics of chromatin SUMOylation during cell fate changes, we used a human B-cell precursor leukemia BLaER1 cell line containing an E2-inducible CEBPA construct whose induction results in macrophage transdifferentiation in 7 days (Fig. 1A) [17]. The transdifferentiation process has been reported to occur in two major transitions, first between 0 and 12 h, followed by a later one from 48 to 72 h [38]. We hypothesized that SUMOylation would be pivoting cell fate processes especially during the early stages, and thus focused on the early timepoints at 0, 6 and 24 h post-E2-induction. We mapped the genome-wide chromatin SUMO landscape by performing ChIP-seq with anti-SUMO2/3 antibody and examined the dynamics of chromatin SUMOylation by categorizing the SUMO2/3 binding sites into unique and shared peaks between timepoints. Intriguingly, chromatin SUMOylation strikingly increased after induction of transdifferentiation. Although a sizeable portion of peaks were already present at 0 h (clusters C1–C4, 19,525 peaks), the vast majority of peaks were only present after induction of transdifferentiation (C5–C7, 67,724 peaks) (Fig. 1B). The majority of SUMO2/3 peaks in all clusters fell within intergenic or intronic regions, suggesting occupancy at gene regulatory regions, such as enhancers (Supplementary Figure S1). The largest population among the SUMO2/3 binding sites present already prior to E2-induction were those shared across all timepoints (C4, 12,616 peaks). Sizeable clusters were also unique to 6 h (C5, 26,670 peaks), 24 h (C7, 17,046 peaks) or shared between these two later timepoints (C6, 24,008 peaks) (Fig. 1B). SUMO2/3 was most enriched in C4 peaks, closely followed by C6 peaks (Fig. 1C, top panel). On the other hand, chromatin accessibility, as measured by ATAC-seq, was highest in C6, followed by C5 and C7 (Fig. 1C, bottom panel), indicating that chromatin accessibility and SUMOylation increase in parallel after induction of transdifferentiation. Spearman correlation analyses of SUMO2/3 ChIP-seq and ATAC-seq read coverages in these SUMO2/3 binding clusters also pointed to strong links between SUMO2/3 occupancy and chromatin accessibility (Supplementary Figure S2).

De novo motif analysis of the binding site clusters revealed distinct, biologically relevant profiles for chromatin sites bound by SUMO2/3 at early stages of the lineage reprogramming. Sites with constant SUMO2/3 occupancy (C4) were enriched with motifs for GATA5, ETS:E-box and SPDEF (an ETS-type motif). Binding sites unique to 6 h (C5) had NFATC2 and HOXB9 motifs, and the sub region III of the downstream core element (DCE), a transcription core promoter sequence. Intriguingly, C6 sites that showed prominently induced SUMO2/3 binding, harbored CEBP:AP1, a composite motif for CEBP and AP-1 family heterodimers, that have been reported to potently direct monopoiesis [39]. Additionally, C7 regions had binding motif for SPIB, a TF closely related to PU.1, which is capable of rescuing macrophage development in the absence of PU.1 [40] (Fig. 1D). Together these results indicate that chromatin SUMOylation status and SUMOylation target regions dynamically change during lineage reprogramming, and that chromatin SUMOylation likely influences the binding of key TFs, such as CEBPA and PU.1, central for macrophage differentiation.

### Chromatin-bound proteins become differently SUMO2/3-associated upon lineage conversion

**Fig. 2:**
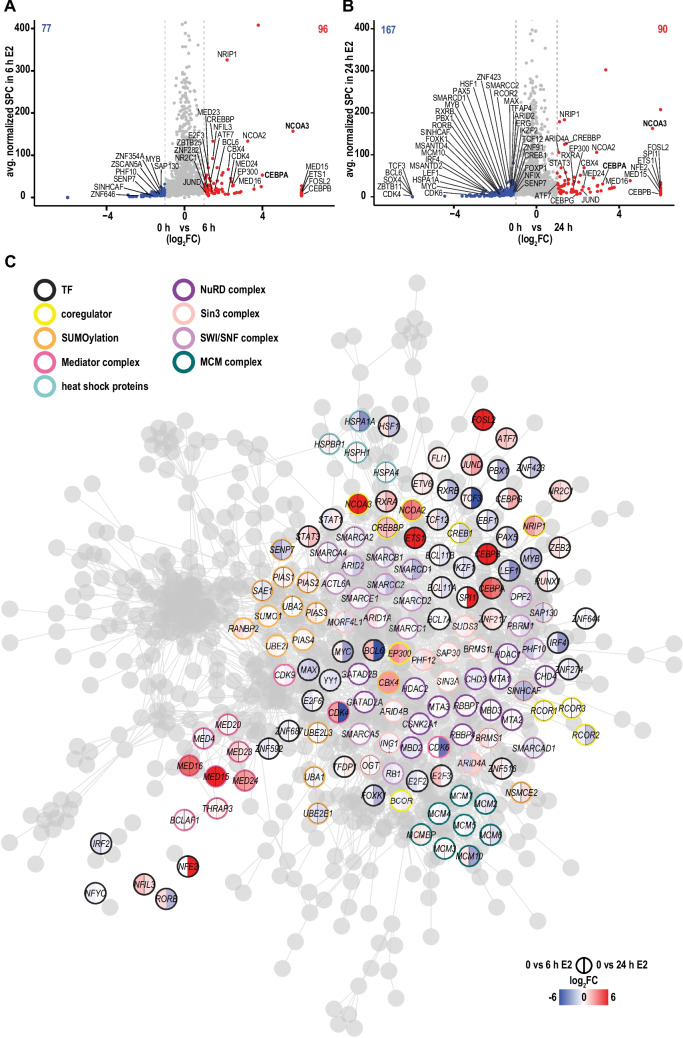
Proteins associated with chromatin-bound SUMO2/3 change upon initiation of lineage conversion. Changes in proteins associated with chromatin-bound SUMO2/3 at 6 h **A** and 24 h **B** after induction of transdifferentiation. Scatterplots depict the SUMO2/3-associated chromatome (FDR < 0.05 in at least one timepoint) in indicated comparisons. Proteins with increased SUMO2/3 association (log_2_[6 h or 24 h E2/0 h E2] > 1) are depicted in red, and proteins with reduced SUMO2/3 association (log_2_[6 h or 24 h E2/0 h E2] < −1) in blue. Y-axis represents average bait-normalized SPC values in the indicated timepoint. CEBPA and NCOA3 are bolded. Numbers in upper corners of the plots denote the number of proteins with increased (red) or reduced (blue) SUMO2/3 association. **C** SUMO2/3 chromatome is a biologically connected protein network (PPI enrichment value 1.0 × 10^–16^). Chromatome members with a STRING interaction score ≥ 0.9 are depicted; selected protein groups are annotated and log_2_FC is shown as indicated. Other proteins in the network are shown in grey

Since genomic localization and intensity of SUMO2/3 displayed dynamic changes during the early stages of transdifferentiation, we next complemented these chromatin landscape data by employing chromatin-directed proteomics (RIME) to map the chromatin-associated protein network (chromatome) of SUMO2/3 at the same timepoints as our ChIP-seq analyses. We identified a total of 1,234 (FDR < 0.05) SUMO2/3-associated proteins across all timepoints. Three quarters (74.4%) of the SUMO2/3 chromatome proteins have previously been found to be SUMOylated [41] (Supplementary Table ST1), strongly suggesting that their residency in the chromatome is largely derived from their covalent modification by SUMO2/3. Although most of the chromatome members did not significantly respond to the initiation of lineage conversion, 337 proteins were differentially associated (|log_2_FC|> 1) with chromatin-bound SUMO2/3 post-induction. Similar numbers of the latter showed increased (UP) or decreased (DN) association with SUMO2/3 during transdifferentiation: At 6 h, 96 proteins were more strongly, and 77 proteins more weakly associated with SUMO2/3 compared to 0 h. When comparing 24 h to the uninduced state, the respective numbers were 90 and 167 (Fig. 2A, B). We interpret this relatively balanced response to the induction of lineage conversion to indicate that SUMOylation of chromatin-associated proteins is a highly regulated and dynamic process.

Overall, a total of 103 TFs were identified in the SUMO2/3 chromatome across all timepoints (FDR < 0.05) (Supplementary Table ST1). Notably, the key driver of transdifferentiation, CEBPA, and its close relatives CEBPB and CEBPG (to a lesser degree) displayed increased SUMO2/3 association after induction of transdifferentiation (Fig. 2A–C), supporting the hypothesis that CEBPA chromatin occupancy is modulated by SUMOylation. Among other more strongly SUMO2/3-associated proteins post-induction were TFs ETS1, PU.1 (SPI1), and STAT3 that have important roles in macrophage development and polarization, as well as AP-1 family members ATF7, FOSL2, and JUND, that also participate in control of cell differentiation. Conversely, the TFs BCL6, ERG, FOXP1, IKZF2, IRF4, LEF1, PAX5, TCF3 and TCF12 with key roles in lymphocyte-related transcriptional programs as well as other TFs MYB, MYC and SOX4 displayed reduced SUMO2/3 association (Fig. 2A, B).

We complemented our RIME data with abundance analysis of chromatin-residing proteins (i.e. RIME input samples) using PAC (see Materials and Methods). PAC detected a total of 5,418 proteins. At 6 h post-induction, no significant changes (|log_2_FC|> 1, FDR < 0.05) in protein abundance were observed, while at 24 h after induction, 124 and 101 proteins displayed increased and decreased abundances, respectively, compared to 0 h (Supplementary Figure S3A, B; Supplementary Table ST2). Of SUMO2/3 chromatome members, 89% (1,099 proteins) were also detected with PAC; 40 of these displayed significant changes in abundance at 24 h compared to 0 h (|log_2_FC|> 1, FDR < 0.05). Comparison of RIME and PAC data indicated that 24 proteins had concordant changes in their SUMO2/3 association and total abundance, i.e. both were increased or decreased at 24 h compared to 0 h (|log_2_FC|> 1 and FDR < 0.05 in both). Of these, only five were TFs: SPI1 showed increase in both abundance and SUMO2/3 association, while ERG, IRF4, LEF1 and TCF3 displayed decrease in both respects (Supplementary Figure S3C, D). Notably, the abundance of CEBPA decreased at 24 h compared to 0 h, which indicates that CEBPA-SUMO2/3 association indeed enhances upon transdifferentiation. These data together suggest that only a relatively modest portion of changes in the SUMO2/3 association of proteins can be attributed to changes in their chromatin abundance.

NCOA3 (SRC3) along with another coactivator family member NCOA2 (SRC2) were among the notable, most strongly SUMO2/3-associated proteins both at 6 h and 24 h post-induction (Fig. 2A, B). Activated TFs, such as nuclear receptors, NF-kB, STAT family members, MEF2C, and CEBPA recruit these proteins that act as platforms bridging activated TFs to enzymatic coactivators/signal integrators, such as p300/CBP (EP300 and CREBBP) [42, 43]. Interestingly, the latter histone acetyltransferases were also found among the more strongly SUMO2/3-associated proteins after the induction of transdifferentiation (Fig. 2A, B). In line with the notion that the TF-NCOA-p300/CBP complex further recruits Mediator complex, MED15, −16, −23, and −24 subunits of the Mediator tail module were found among the more strongly SUMO2/3-associated chromatome (Fig. 2A, B). The tail module of Mediator is especially important for facilitating interactions between TFs and the core Mediator complex, which subsequently relays signals to the RNA Pol II machinery [44]. Other subunits of the Mediator complex (MED4 and −20) and closely associated proteins (BCLAF1, THRAP3) were also in the SUMO2/3 chromatome although their association with SUMO2/3 was not altered during the early stages of transdifferentiation (Fig. 2C). Members of the SWI/SNF complex (ARID2, SMARCC2, SMARCD1) displayed reduced SUMO2/3 association after 24 h of E2 induction (Fig. 2B, C). In addition to the SWI/SNF subunits, the chromatome included chromatin remodeler NuRD and Sin3 complex subunits (Fig. 2C).

Expectedly, proteins participating in the SUMOylation machinery, e.g. SAE1 and PIAS1-4, were also identified, although they interestingly did not display changes in their SUMO2/3 association, apart from SENP7 whose SUMO2/3 association attenuated after induction and CBX4 whose association was enhanced (Fig. 2B, C). Overall, the SUMO2/3 chromatome, that included also several other transcriptional coregulators (e.g. BCOR and RCOR1–3), had a PPI enrichment value of 1.0 × 10^–16^, indicating the identified members of the chromatome form a biologically connected network, and not merely an arbitrary collection of proteins.

Together, these results suggest that SUMOylation on chromatin is dynamically involved in early transdifferentiation, potentially playing a central role in modulating TF activity and chromatin-associated protein networks. However, the altered SUMO2/3 association of some proteins, e.g. increased SUMO2/3 association of a macrophage-related TF PU.1 and reduced association of IRF4, a TF linked to lymphocyte identity, may reflect changes in their abundance during the cell fate change from B-cells to macrophages.

### Extensive convergence between CEBPA and SUMO2/3 chromatin-associated networks

To further explore the regulatory role of chromatin SUMOylation in CEBPA-driven lineage conversion, we analyzed the chromatome of CEBPA during early transdifferentiation. Across all timepoints, we identified a total of 313 CEBPA-associated proteins (FDR < 0.05). Notably, approximately 65% (202 proteins) of these were shared with the SUMO2/3 chromatome (FDR < 0.05 in at least one timepoint), indicating a substantial convergence between SUMOylated chromatin environments and CEBPA-mediated transcriptional regulation (Supplementary Table ST3).

**Fig. 3:**
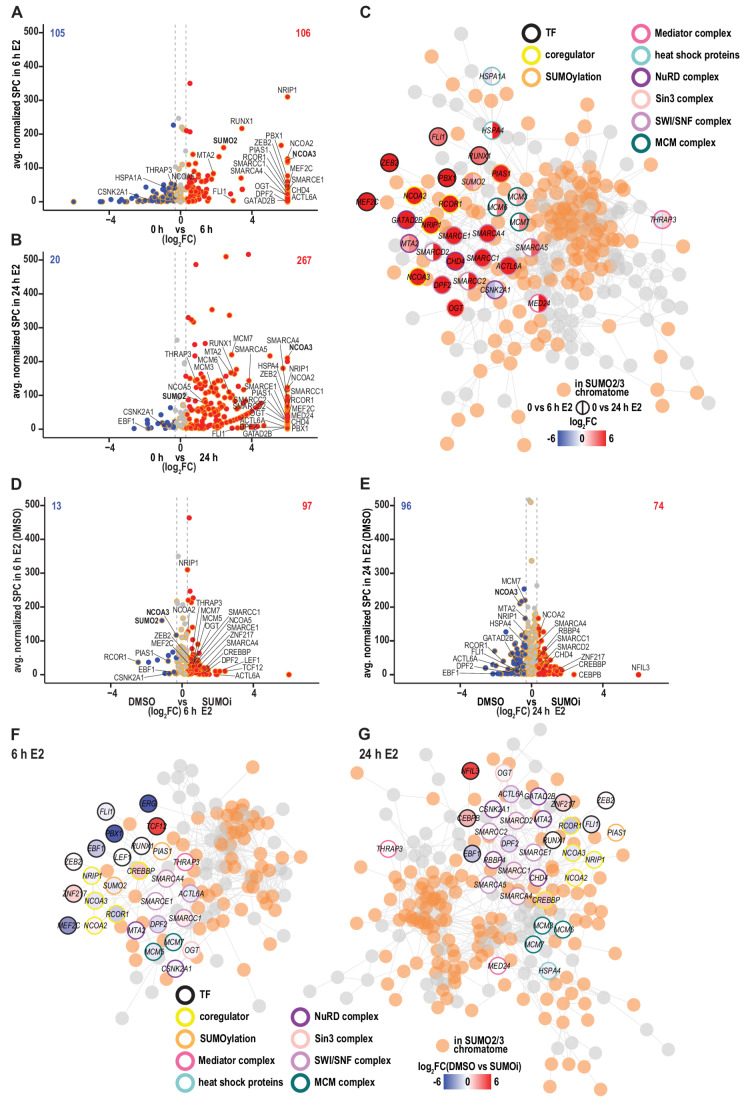
CEBPA’s chromatin protein network is intertwined with and modulated by SUMOylation. **A** and **B** Changes in proteins associated with chromatin-bound CEBPA at 6 h (A) and 24 h (B) after induction of transdifferentiation. Scatterplots depict the CEBPA-associated chromatome (FDR < 0.05 in at least timepoint) in indicated comparisons. Proteins with increased CEBPA association (log_2_[6 h or 24 h E2/0 h E2] > 0.3) are depicted in red, and proteins with reduced CEBPA association (log_2_[6 h or 24 h E2/0 h E2] < −0.3) in blue. Y-axis represents average bait-normalized SPC values at the indicated timepoint. NCOA3 and SUMO2 are bolded. Numbers in upper corners of the plots denote the number of proteins with increased (red) or reduced (blue) CEBPA association. In all panels, proteins found also in SUMO2/3 chromatome (FDR < 0.05 in at least one timepoint) are indicated with orange or annotations for selected proteins. **C** TFs, coregulators (e.g. NCOA3), chromatin remodelers etc. in CEBPA chromatome are shared with SUMO2/3 chromatome. Chromatome members with a STRING score ≥ 0.7 are depicted; selected protein groups are annotated and log_2_FC is shown as indicated. Other proteins in the network are shown in grey. **D** and **E** SUMOylation modulates the composition of CEBPA chromatome. Scatterplots show the effect of SUMOi on CEBPA chromatome at 6 h (D) or 24 h (E) after induction of transdifferentiation. Proteins with increased CEBPA association upon SUMOi (log_2_[SUMOi/DMSO] > 0.3) are depicted in red, and proteins with reduced CEBPA association (log_2_[SUMOi/DMSO] < −0.3) in blue. Y-axis represents average bait-normalized SPC values in DMSO samples. NCOA3 and SUMO2 are bolded. Numbers in upper corners of the plots denote the number of proteins with increased (red) or reduced (blue) CEBPA association. **F** and **G** Networks corresponding to **D** and **E**, respectively. Chromatome members with a STRING interaction score ≥ 0.7 are depicted; selected protein groups are annotated and log_2_FC is shown as indicated. Other proteins in the network are shown in grey

At 6 h post-induction, 211 proteins showed differential association with CEBPA (|log_2_FC|> 0.3); about half of them (106) displaying increased association and half (105) reduced association. Coregulators, such as NCOA2, NCOA3 and RCOR1, and SWI/SNF complex subunits displayed increased association with CEBPA (Fig. 3A), which is in line with active coregulator recruitment and transcriptional complex assembly by CEBPA. By 24 h, the CEBPA chromatome expanded further, with 267 proteins displaying increased association and only 20 decreased association compared to uninduced cells. Most proteins (~ 97%) that had exhibited increased CEBPA association at 6 h continued to do so at 24 h. For example, the above coregulators and TFs FLI1, MEF2C, PBX1, RUNX1 and ZEB2 continued to display enhanced association with CEBPA. Among the newly recruited proteins in the CEBPA chromatome at 24 h was the Mediator subunit MED24, that was also increasingly SUMO2/3-associated during early transdifferentiation. Additional SWI/SNF complex members (SMARCC2, SMARCD2) were also present, along with those found in the chromatome already at 6 h (SMARCA4, SMARCC1, SMARCE1) (Fig. 3B, C). These results are likely to reflect expanded chromatin occupancy and transcriptional regulation by CEBPA from 6 to 24 h post-induction.

### SUMOylation modulates the protein network around chromatin-bound CEBPA

To assess the influence of SUMOylation on the association of proteins with chromatin-bound CEBPA, we employed a selective SUMO E1 inhibitor ML-792 (SUMOi) to inhibit SUMOylation in BLaER1 cells. Immunoblotting with the SUMO2/3 antibody confirmed markedly reduced SUMOylation level (a hypoSUMOylated cellular state) remaining until 72 h post-induction after 24 h pre-treatment with 100 nM ML-792, a concentration that did not cause substantial decline in BLaER1 cell proliferation as assessed by MTS assays (Supplementary Figure S4A, B). Before induction (0 h), SUMOi slightly altered the CEBPA chromatome, with 35 proteins displaying decreased association and 9 increased association (Supplementary Table ST3). At 6 h after induction, SUMOi effect was enhanced but the pattern reversed: 97 proteins displayed increased and 13 decreased association (Fig. 3D). By 24 h, the effect of SUMOi was more balanced, with similar numbers of proteins exhibiting increased (74) and decreased (96) association (Fig. 3E). Reduced association of SUMO2 with CEBPA provided additional proof for the efficiency of SUMOi (Fig. 3D, F).

On the level of individual proteins, the effect of SUMOi was nuanced. For example, NCOA3, EBF1 and RCOR1 displayed consistently weakened association in response to SUMOi (Fig. 3D, E), suggesting that their recruitment to CEBPA chromatome is SUMOylation-enhanced. On the other hand, CREBBP and SWI/SNF complex members SMARCA4, SMARCC1 and SMARCE1 displayed increased association, implying that SUMOylation may hamper their association with CEBPA (Fig. 3D). Some proteins, such as CEBPB, SWI/SNF complex member ACTL6A, and GATAD2B, a core subunit of NuRD complex, exhibited induction time-dependent responses to SUMOi (Fig. 3D, E), suggesting a transdifferentiation phase-dependent regulation by SUMOylation. Together, these data imply that SUMOylation modulates CEBPA’s associations with its chromatin partners in a protein-selective fashion.

### Chromatin occupancy of CEBPA and SUMO2/3 during the early phases of lineage conversion

**Fig. 4:**
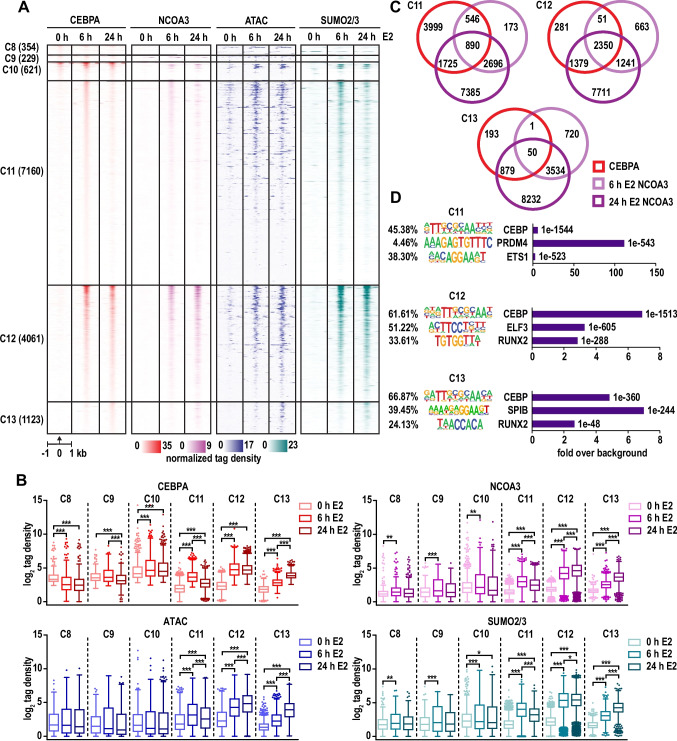
CEBPA occupies distinct chromatin regions with SUMO2/3 and NCOA3 at the early phases of lineage conversion. **A** CEBPA occupancy on chromatin occurs in waves. Heatmaps of CEBPA, NCOA3, SUMO2/3 occupancy on chromatin and chromatin accessibility at CEBPA-binding sites during early stages of transdifferentiation. C8: CEBPA-binding sites unique to 0 h; C9: shared between 0 and 6 h; C10: shared between 0–24 h; C11: unique to 6 h; C12: shared between 6 and 24 h; C13: unique to 24 h. **B** Boxplots of CEBPA, NCOA3 and SUMO2/3 ChIP-seq and ATAC-seq signal (tag density) in the CEBPA-binding clusters C8–13 depicted in (A). **C** Venn diagrams of the overlap between indicated CEBPA peak cluster and NCOA3 peaks at 6 h E2 and 24 h E2. **D** Top three de novo motifs for C11–C13, showing enrichment as fold over background and respective *p*-values at the end of the bars. Percentages indicate the percentage of CEBPA-binding sites with motif. Statistical significance for boxplots was calculated with One-way ANOVA with Bonferroni post hoc test, with asterisks denoting statistical significance: * = *p* < 0.05, ** = *p* < 0.01, *** = *p* < 0.001

As our results indicated that SUMO2/3 and CEBPA co-occupy chromatin, we next moved on to map the CEBPA cistrome similarly as for SUMO2/3 above. Although there were some peaks present at 0 h (C8, 354 peaks) and persisting peaks shared between 0 and 6 h (C9, 229 peaks) or across all timepoints (C10, 621 peaks), the vast majority of peaks occurred only after the induction of transdifferentiation (12,344 peaks), consistent with the model of activated CEBPA binding (Fig. 4A, B). The latter binding sites were further categorized into transient peaks present only at 6 h (C11, 7,160 peaks), later peaks at 24 h (C13, 1,123 peaks) or sustained peaks that were induced at 6 h and remained at 24 h (C12, 4,061 peaks). Chromatin accessibility was increased (compared to uninduced cells at 0 h) in these post-induction clusters in parallel with the occupancy of CEBPA (Fig. 4A, B).

Since NCOA3 was identified among the strongest SUMO2/3-associated chromatin partners of CEBPA and reported to act as a key regulator of adipocyte differentiation program, cooperating with CEBPA [45], we also investigated its chromatin occupancy. NCOA3 signal was also present in the CEBPA binding sites (Fig. 4A, B). The NCOA3 signal generally reflected the dynamics of CEBPA signal, with NCOA3 occupancy peaking in C11 at 6 h, and in C12 and C13 at 24 h (Fig. 4B). Chromatin SUMOylation followed a likewise pattern, with the strongest signal in C12, reflecting the results of SUMO2/3 binding site analysis (Fig. 1B) where chromatin SUMOylation was the most prevalent at the 6 h and 24 h sustained shared sites (C6). Closer inspection of the overlap of CEBPA and NCOA3 peaks revealed the highest portion of co-occupied sites in C12, with 93% of CEBPA peaks overlapping with NCOA3 peak (3,780 out of 4,061 sites, Fig. 4C).

Correlation analyses of ChIP-seq and ATAC-seq read coverages in C8-13 largely reflected parallel CEBPA, SUMO2/3 and NCOA3 chromatin occupancy and chromatin accessibility, with moderate to strong correlations between all the ChIP-seq and ATAC-seq signals at 6 h and 24 h after induction (Supplementary Figure S5). Read coverages across the genome also displayed correlation between chromatin accessibility and CEBPA, SUMO2/3 and NCOA3 occupancy, with CEBPA and SUMO2/3 especially clustering closely together (Supplementary Figure S6).

The top de novo binding motif across all post-induction clusters (C11-13) was, expectedly, for CEBP factors. However, beyond this shared motif, each cluster harbored binding motifs for distinct TFs: C11 featured motifs for PRDM4 and ETS1, C12 for ELF3 and RUNX2, and C13 for SPIB in addition to RUNX2 (Fig. 4D). These results suggest that at early phases of the transdifferentiation process, CEBPA binds to divergent chromatin regions harboring diverse binding motifs, co-operating with different TFs at different phases of the process. NCOA3’s chromatin occupancy and chromatin SUMOylation mirror that of CEBPA, suggesting functionally relevant interactions.

### SUMOi enhances CEBPA chromatin occupancy

**Fig. 5:**
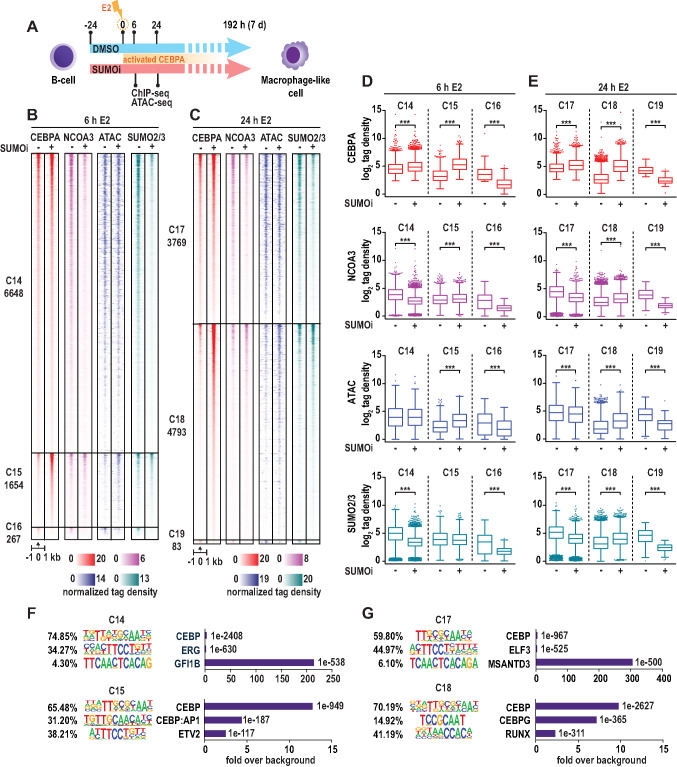
SUMOi augments CEBPA chromatin occupancy. **A** Experimental setup and timeline with control (DMSO) and SUMOi treatments. **B** and **C** CEBPA-binding sites on chromatin in response to SUMOi at 6 h and 24 h after induction of transdifferentiation, respectively. Binding sites are categorized into moderately SUMOi-affected CEBPA-binding sites (C14 and C17, difference between DMSO and SUMOi < 3-fold), clearly SUMOi-augmented CEBPA-binding sites (C15 and C18, difference between DMSO and SUMOi > 3-fold) and SUMOi-impaired sites (C16 and C19, difference between DMSO and SUMOi > 3-fold). Heatmaps depict CEBPA, NCOA3, SUMO2/3 and ATAC occupancy in the clusters. **D** Boxplots of CEBPA, NCOA3 and SUMO2/3 ChIP-seq and ATAC-seq signal (tag density) in C14–16. **E** Boxplots of CEBPA, NCOA3 and SUMO2/3 ChIP-seq and ATAC-seq signal (tag density) in C17–19. **F** and **G** Top three de novo motifs for C14–15 and C17–18, respectively, showing enrichment as fold over background and respective p-values at the end of the bars. Percentages indicate the percentage of CEBPA-binding sites with motif. Statistical significance for boxplots was calculated with One-way ANOVA with Bonferroni post hoc test, with asterisks denoting statistical significance: * = *p* < 0.05, ** = *p* < 0.01, *** = *p* < 0.001

We next investigated how SUMOi affects CEBPA and NCOA3 chromatin binding by using ChIP-seq (Fig. 5A). As shown in Fig. 5, hypoSUMOylation generally enhanced CEBPA binding. Clustering of CEBPA binding sites according to the effect of SUMOi at 6 h of transdifferentiation revealed that SUMOi had a moderate effect (difference between DMSO and SUMOi < 3-fold) on CEBPA binding on 6,648 sites (C14), while 1,654 sites (C15) displayed substantially augmented CEBPA binding (> 3-fold difference). At 24 h, the number of the latter SUMOi-augmented sites (C18, 4,793 sites) exceeded that of the moderately affected sites (C17, 3,769 sites) (Fig. 5B, C). At 6 h, 843 of the SUMOi-augmented peaks in C15 were specific to SUMOi, while 811 were also present in DMSO (Supplementary Figure S7A). Interestingly, later, at 24 h, the portion of SUMOi-specific peaks in C18 dramatically increased, with 3,703 peaks detected only in SUMOi-treated cells and 1,090 peaks in both conditions (Supplementary Figure S7B). This indicates that SUMOi both stabilizes CEBPA and facilitates its binding to new chromatin sites. SUMOi also affected a minuscule number of sites in the opposite manner, i.e. reduced CEBPA binding (C16, 267 sites and C19, 83 sites at 6 h and 24 h, respectively) (Fig. 5B, C). Chromatin accessibility increased in C15 and C18, but decreased in C14 and C17, in response to SUMOi (Fig. 5D, E). Interestingly, the effect of SUMOi on NCOA3 chromatin occupancy largely paralleled its effect on chromatin accessibility (Fig. 5D, E). In keeping with the immunoblotting results, chromatin SUMO2/3 signal was clearly decreased in C14 and C17 (as well as in C16 and C19), whereas in C15 SUMO2/3 signal did not clearly respond to SUMOi, and in C18 it even displayed slight elevation after SUMOi (Fig. 5B–E). This suggests that SUMO dynamics are likely to be differently regulated in different chromatin regions.

As above for temporal CEBPA cistrome analysis, the top de novo binding motif in all clusters was for CEBPA, although the portion of sites enriched for the motif slightly varied between the clusters (59.80–74.85%). However, other binding motifs in the top three differed considerably across the clusters. In C14, the motif with the second lowest *p*-value was for ERG (34.27% of sites), while the third one was for GF1B (4.30% of the sites). Interestingly, ERG was also found in the SUMO2/3 chromatome. In C15, CEBP:AP1 composite motif (31.20% of the sites) and ETV2 motif (38.21%) were the respective ones (Fig. 5F). At 24 h, the binding sites again displayed different binding motif profiles, with C17 being enriched for ELF3 and MSANTD3 motifs, and C18 for CEBPG and RUNX motifs (Fig. 5G). A TF closely related to ELF3 (ELF1) was identified as a SUMO2/3-associated protein on chromatin (Supplementary Table ST1), and CEBPG increasingly so, at 24 h (Fig. 2B, C), suggesting their SUMO-modulated cooperative role with CEBPA during transdifferentiation.

Collectively, these data imply that SUMOylation may buffer CEBPA binding or access to chromatin and modulate recruitment of NCOA3. The former may partly be due to SUMOylation participating in the forming of repressive chromatin complexes [12]. Motif enrichment analyses also point to the possibility that SUMOylation programs CEBPA to bind to alternative sites, co-operating with different TFs.

### SUMOi alters transcriptional profiles during lineage reprogramming

**Fig. 6:**
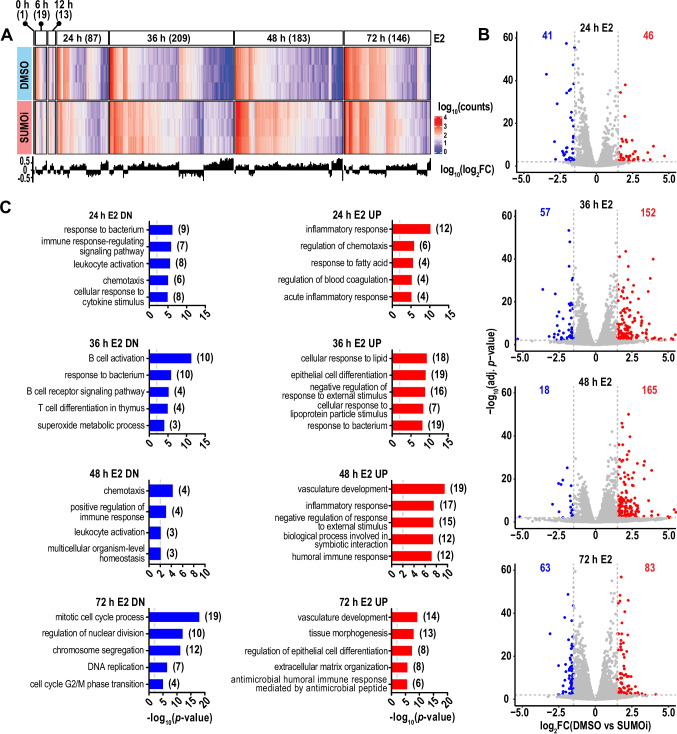
SUMOi alters transcriptional profiles during transdifferentiation. **A** Heatmap of RNA-seq conditional comparisons, depicting DMSO vs SUMOi DEGs, (|log_2_FC|= 1.5, adj. *p*-value < 0.01) within each timepoint. Numbers in parentheses denote the number of DEGs in each timepoint. Histogram below the heatmap shows the log_10_-transformed log_2_FC value for the gene above it. **B** SUMOi chiefly upregulates gene expression at 36 h and 48 h. Volcano plots of DEGs at 24–72 h after induction, depicting log_2_FC and -log_10_ adj. *p*-values for pairwise comparisons between treatments. Numbers indicate the number of genes that were upregulated (red, log_2_FC > 1.5, -log_10_ adj. *p*-value > 2) and downregulated (blue, log_2_FC < −1.5, -log_10_ adj. *p*-value > 2) in response to SUMOi. **C **SUMOylation dynamically controls gene programs related to B-cell and macrophage identities. Bar plots of Metascape pathway analyses of SUMOi up- or downregulated genes at the same timepoints as **B**. Grey line indicates -log_10_ transformation of significance threshold (adj. *p*-value < 0.01) after built-in Benjamini–Hochberg multiple correction in Metascape

We next employed RNA-seq to investigate transcriptomic profiles during the early and intermediate stages of transdifferentiation (0–72 h). Principal component analysis of all the RNA-seq samples at 0, 6, 12, 24, 36, 48 and 72 h post-induction in the absence and presence of SUMOi indicated that most variance was temporal, due to progression of the transdifferentiation process. Moreover, pre-treatment with SUMOi did not extensively alter gene expression before the induction of transdifferentiation. However, at 36 h post-induction, gene expression of SUMOi-treated cells started to clearly diverge from control cells (Supplementary Figure S8). Clustering of genes based on conditional comparison (|log_2_FC|≥ 1.5, adj. *p*-value < 0.01) within each timepoint also confirmed that SUMOi treatment by itself did not lead to major transcriptomic changes before the induction of lineage conversion, as only one gene (*JCHAIN*) was affected. Even after 6 h and 12 h, only a modest number of genes, 19 and 13, respectively, showed significantly different expression between the two conditions. However, this does not rule out more modest changes in expression. At 24 h, the number of SUMOi-affected genes markedly increased, with 87 genes showing differential responses. At later timepoints, the number of DEGs further increased, with 36 h timepoint showing the largest number of affected genes (209) (Fig. 6A, Supplementary Table ST4).

Based on Metascape pathway analysis, SUMOi both up- and downregulated processes related to inflammatory and immune responses. SUMOi repressed genes involved in leukocyte and B-cell activation, and B-cell receptor signaling pathway between 24–48 h after induction. The upregulated gene programs at 24 h and 36 h included lipid metabolism-related processes (response to fatty acid and cellular response to lipid, respectively), and tissue remodeling, vasculature development, tissue morphogenesis, and extracellular matrix organization during later stages, at 48 h and 72 h (Fig. 6C, Supplementary Table ST5). Notably, downregulated processes at 72 h heavily featured those involved in cell cycle and DNA replication control, suggesting that SUMOylation modulates also the progress of cell cycle, which is inherently interconnected with cell differentiation (Fig. 6C).

### Association of SUMOi-affected gene expression with chromatin SUMOylation and CEBPA binding

**Fig. 7:**
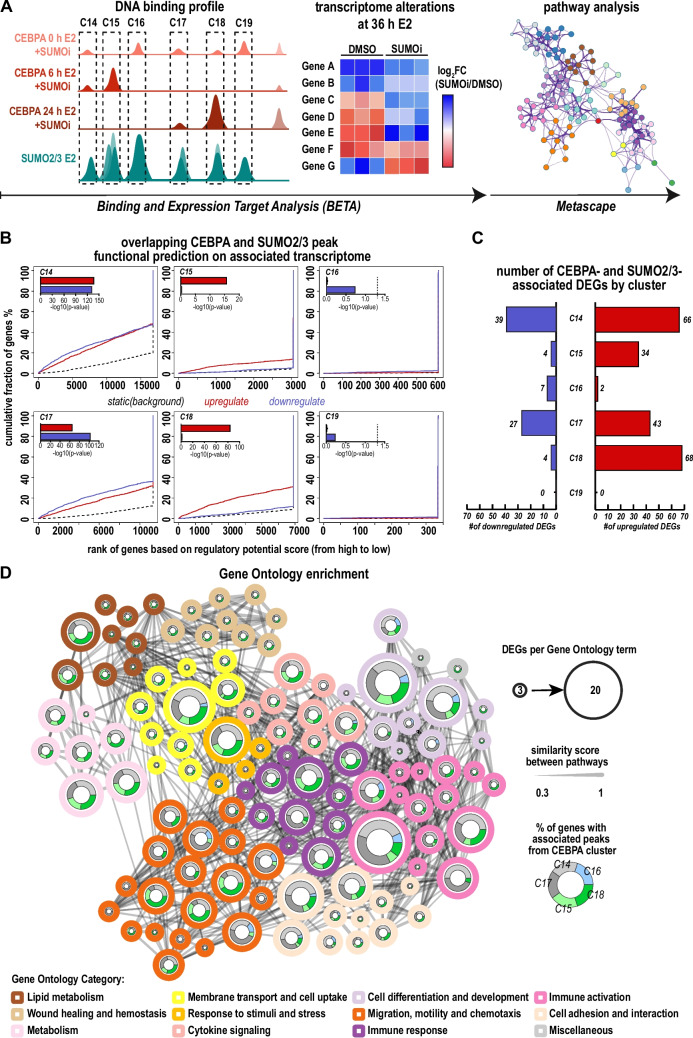
Differentially SUMOi-affected CEBPA-binding sites have divergent regulatory functions. **A** Overview of chromatin binding and gene expression data integration analysis. Differently SUMOi-affected CEBPA clusters C14–C19 were overlapped with SUMO2/3 clusters C1–C7 and integrated with transcriptomic changes at 36 h timepoint using BETA, followed by pathway analysis in Metascape. **B** Functional prediction of CEBPA binding in indicated clusters with overlapping SUMO2/3 peak. SUMOi-augmented binding sites (C15 and C18) are predominantly upregulatory. Background of non-regulated genes is indicated with dashed black line, cumulative CEBPA activation function with red line and cumulative CEBPA repression function with blue line. Genes are cumulated by the rank based on the regulatory potential score from high to low. *P*-values from the Kolmogorov–Smirnov test for difference to background are indicated in the top left corner bar plots in each set. Dotted line denotes significance threshold (*p*-value < 0.05). **C** Number of CEBPA- and SUMO2/3-associated DEGs by CEBPA-binding cluster. **D** Enrichment of all CEBPA- and SUMO2/3-associated genes in Gene Ontology categories. SUMOi-augmented CEBPA-binding sites are particularly associated with genes related to lipid metabolism

To gain more mechanistic insight, we integrated SUMO2/3 and CEBPA ChIP-seq signals with transcriptome changes. First, differently SUMOi-affected CEBPA clusters C14–C19 were overlapped with SUMO2/3 clusters C1–C7. A substantial fraction (36–75%) of CEBPA peaks within the CEBPA binding clusters overlapped with a SUMO2/3 peak, primarily with C6 binding sites that were induced in a sustained fashion upon lineage conversion (Supplementary Figure S9A, B). The SUMO2/3-overlapping CEBPA peaks were subsequently analyzed by using BETA to predict their regulatory potential on transcriptome. BETA predicts direct target genes and infers the overall regulatory role of TF by integrating binding data and gene expression profiles. Specifically, it estimates the contribution of individual TF binding sites (here, CEBPA) within a certain distance from target gene TSS to the regulatory potential using a distance-weighted function, and it combines this with the differential gene expression upon TF binding. To account for transcriptional delay relative to chromatin binding, we used the 36 h post-induction timepoint DEGs showing at least one CEBPA-binding site within 100 kb from a TSS (Fig. 7A). Based on BETA analysis, SUMO2/3-overlapping CEBPA binding sites in C14 and C17 only moderately affected by SUMOi are predicted to nearly equally up- and downregulate target genes (Fig. 7B). In contrast, the substantially SUMOi-enhanced CEBPA binding sites with SUMO2/3 peaks in C15 and C18 showed practically solely upregulatory potential, with the potential increasing from 6 h (C15) to 24 h (C18). These differences are in line with DEG counts (Fig. 7C).

We subsequently examined the protein level interactions of CEBPA- and SUMO2/3-associated DEGs in STRING database. More than 60% of the DEG-coded proteins had at least one interaction with another protein in the set (Supplementary Figure S10). Additionally, the network of interactions produced significantly more interactions than expected for a set of similar number of proteins (PPI enrichment value 1.0 × 10^–16^), underscoring their biological connections and relations with each other. Therefore, we explored pathway enrichment among them. This led to identification of several B-cell and macrophage-related pathways and categories, with the substantially SUMOi-augmented CEBPA binding sites showing association particularly with lipid metabolism pathways (Fig. 7D). *PPARG*, *CD36* and *APOE*, all displaying markedly higher expression and CEBPA binding in response to SUMOi, are notable examples of genes in lipid metabolism, having also important roles in inflammatory responses macrophages (Supplementary Figure S11). This suggests that SUMOi can also propel lineage conversion by inducing metabolic reprogramming towards macrophage phenotype.

### SUMOi accelerates the appearance of macrophage transcriptomic programs

**Fig. 8:**
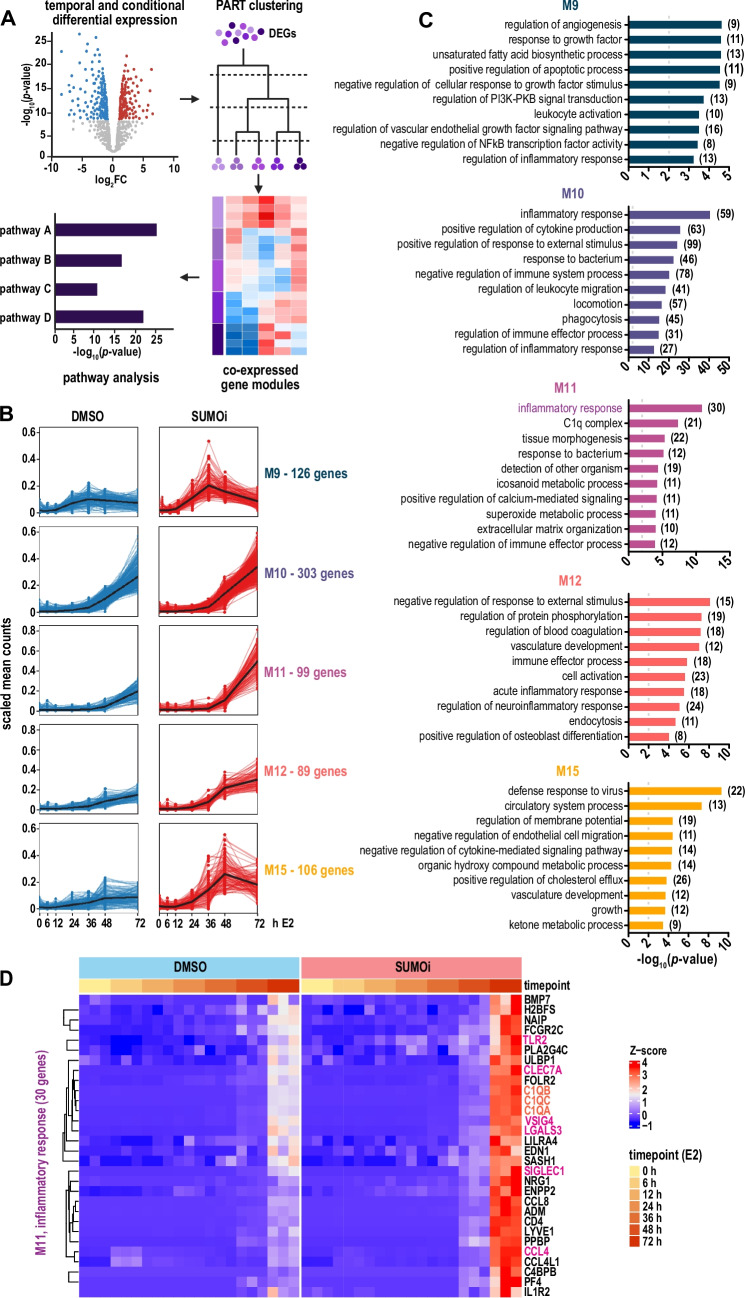
SUMOi accelerates transcriptional reprogramming. **A** Overview of co-expressed gene module analysis. PART-based clustering was used to divide temporal and conditional DEGs into co-expressed modules, which were subsequently subjected to pathway analysis. **B** Expression trajectories for five gene modules that display different expression patterns in DMSO and SUMOi. **C** Metascape GO-term analyses of genes in the five modules depicted in **B**. Grey line indicates -log_10_ transformation of significance threshold (adj. *p*-value < 0.01) after built-in Benjamini–Hochberg multiple correction in Metascape. **D** Heatmap of genes included in the top pathway of M11 (inflammatory response), which includes several macrophage markers (denoted in pink) and complement components (denoted in orange)

To further examine how SUMOi affects the transcriptomic programs during lineage reprogramming, we utilized PART-based clustering that considers both temporal and conditional changes to categorize DEGs into co-expressed modules (Fig. 8A). This approach revealed 15 modules (M) with different expression profiles during the first half of transdifferentiation (until 72 h; Supplementary Figure S12A, B). Out of these, 5 modules (M9–12 and M15), were particularly interesting, displaying different trajectories in control and SUMOi. Reflecting the conditional comparisons above, the trajectories started to differ at later timepoints, with SUMOi having a predominantly upregulatory effect on the modules (Fig. 8B). Metascape analysis showed that all these modules included pathways involved in inflammatory and immune responses, also mirroring the results from conditional comparisons above. The modules M9 and M15 where SUMOi upregulated genes between 24–72 h, additionally included processes related to metabolism and growth (Fig. 8C, Supplementary Table ST6).

We next focused on the M11 module displaying the most differing trajectories and examined more closely the genes involved in the top pathway, inflammatory response. This revealed a stark difference in the expression of many macrophage marker genes, e.g. *CCL4, CLEC7A, LGALS3, SIGLEC1, TLR2 *and* VSIG4*, and complement components, e.g. *C1QA, C1QB *and* C1QC*. All 30 genes included in the inflammatory response pathway were robustly upregulated by SUMOi at 72 h, strongly suggesting that hypoSUMOylation promotes macrophage identity (Fig. 8D). Additionally, analysis of a larger group of macrophage marker genes, both general macrophage markers and those linked to M1 and M2 macrophages, revealed enhanced expression under hypoSUMOylated conditions (Supplementary Figure S13A–C). Collectively, these data indicating dynamic control of gene programs related to B-cell and macrophage identities suggest that SUMOylation helps to maintain B-cell identity, while its inhibition facilitates transdifferentiation. We suggest that in lineage conversion, chromatin SUMOylation is one of the safety mechanisms fine-tuning the pace and fidelity of lineage conversion.

## Discussion

Pharmacological suppression of cellular SUMOylation levels has been shown to facilitate differentiation and other cell fate transitions [3, 13] but the genome-wide dynamics of chromatin SUMOylation and its interplay with master TFs during early phases of lineage conversion have not been explored. Here, we utilized a well-established BLaER1 cell system [17, 38] to study the impact of SUMOylation during human B-cell-to-macrophage transdifferentiation. Prior work by Cossec et al. [3] noted that Ubc9 depletion enhanced macrophage traits in an analogous murine model, but did not characterize chromatin or transcriptional dynamics. Our study demonstrates that chromatin SUMOylation plays a dynamic and gene context-dependent role in CEBPA-driven lineage conversion. Notably, rather than accumulating onto inactive closed chromatin, SUMOylation by SUMO2/3 prominently increased on chromatin regions showing enhanced chromatin openness upon induction of lineage conversion.

We show that CEBPA programs the transdifferentiation process by binding to chromatin in waves, with both transient and more sustained binding events emerging after induction. Chromatin SUMOylation was similarly temporally structured, with distinct chromatin regions accumulating SUMO2/3 in response to the induction of lineage conversion. Fittingly, the temporal pattern of CEBPA chromatin occupancy mirrored the substantial remodeling of the CEBPA chromatin-associated protein network during the early phases of transdifferentiation. Moreover, most members of this network associated with chromatin-bound SUMO2/3, which itself had a large, biologically interconnected network containing several relevant coregulators, chromatin remodelers, Mediator tail module components, and particularly TFs involved in lymphoid and myeloid development. Altogether, these results align with our previous observations of the intertwinement of chromatin protein networks of SUMO2/3 and those of the androgen receptor and the glucocorticoid receptor [16, 18], collectively supporting the notion that SUMOylation accompanies active TFs and associated transcriptional complexes on chromatin and shapes their protein–protein interactions.

SUMOi led to enhanced chromatin binding of CEBPA, reflecting our earlier observations of SUMOi-augmented glucocorticoid receptor cistrome in leukemic B-cells [18]. Interestingly, the substantially SUMOi-enhanced CEBPA binding sites displayed, besides CEBP motifs, distinct binding motif profiles compared to other CEBPA binding sites, implying that SUMOylation facilitates or hinders the crosstalk of CEBPA with its neighboring TFs. This behavior of CEBPA echoes the model proposed by Rosonina, in which SUMOylation affects the binding site selection of TFs by affecting site specificity [46].

In line with these notions, our chromatin proteomic analyses revealed SUMOylation-dependent changes in the composition of CEBPA-associated protein network. For example, SWI/SNF chromatin remodeling complex subunits, including SMARCA4 (BRG1), an essential ATPase subunit of the complex, were increasingly CEBPA-associated as transdifferentiation proceeded, with their association further augmented upon SUMOi. Interestingly, SUMOylation of CEBPA has previously been reported to hamper its interaction with SMARCA4 [47]. On the other hand, NCOA3, a critical coregulator of adipocyte differentiation program and CEBPA [45], showed attenuated association with CEBPA upon SUMOi. Reflecting this, NCOA3 co-occupancy decreased in response to SUMOi on the CEBPA binding sites displaying also reduced SUMOylation. However, on the substantially SUMOi-enhanced CEBPA binding sites where SUMO2/3 level was not reduced by SUMOi, NCOA3 occupancy was not attenuated. This apparent discrepancy between these differentially SUMOi-responsive chromatin sites may reflect several mechanisms. SUMOylation might exert both repressive and permissive effects, depending on the chromatin context and transcriptional state. Such dual functionality has been observed in stress responses, where SUMOylation can either suppress or facilitate transcription depending on the target and cellular conditions [14, 48–50]. Additionally, it raises the possibility that SUMOi may evoke compensatory mechanisms that stabilize already SUMOylated proteins, as it only blocks the activation step (E1) without necessarily affecting pre-existing SUMOylation of proteins, albeit the modification is generally thought to be erased rapidly. Regardless, on account of this relationship between CEBPA, NCOA3 and chromatin SUMOylation, our results are in line with the previous finding that the transcriptional activity of NCOA3 (a.k.a. AIB1) is regulated by SUMOylation [51]. As a limitation of the study, the ER hormone-binding domain in the CEBPA fusion protein used in the BlaER1 system may contribute to coregulator recruitment, so some differences from wild-type CEBPA cannot be excluded.

General transcriptomic profiling revealed that SUMOi had a minimal impact on gene expression in uninduced cells, but it progressively altered transcriptional programs during lineage conversion. In alignment with our previous results in prostate cancer cells and leukemic B-cells, SUMOylation did not solely act as a repressive modification but it both activated and repressed gene expression, depending on the stage of transdifferentiation, contrasting with initial notions [12, 16, 18, 52]. At the end of the late transition phase (occurring between 48–72 h) of B-cell-to-macrophage gene expression [17], SUMOi clearly promoted the expression of macrophage identity genes, while repressing B-cell-associated programs and cell cycle regulators. Clustering analyses further revealed distinct gene modules, enriched for immune response, tissue remodeling and metabolism related genes, that responded to SUMOi in a time-dependent manner. These findings imply that SUMOylation constrains the full activation of macrophage-specific gene programs, acting as a gatekeeper of lineage fidelity and timing. However, assessment of nascent transcription, instead of RNA-seq that primarily uncovers differences in steady-state mRNA levels, could have revealed more immediate and SUMOi-specific effects on transcription/RNA synthesis.

Integration analysis of chromatin binding and transcriptomics data revealed that the substantially SUMOi-enhanced CEBPA binding sites predominantly upregulated gene expression, which aligns with SUMOylation negatively affecting the transcriptional activity of CEBPA [53, 54]. These SUMOi-enhanced CEBPA binding sites were particularly associated with genes related to lipid metabolism pathways. In accordance with the role of the CEBP family, including CEBPA, as a key regulator of lipid metabolism-related differentiation [55], these pathways contain several genes, notably *PPARG*, that also play important roles in the differentiation and activation of macrophages [56, 57]. Our findings suggest that SUMOi may influence the lineage conversion to macrophages partly by promoting metabolic reprogramming.

Taken together, our data support a model in which SUMOylation of chromatin-bound proteins fine-tunes the balance between lineage identity and functional maturation, ensuring orderly progression through the reprogramming trajectory. Rather than occupying inaccessible, inactive chromatin regions, SUMO2/3 is dynamically connected with shaping transcriptional activity and chromatin accessibility during transdifferentiation. SUMOylation can orchestrate the timing and fidelity of cell fate transitions by modulating TF binding, coregulator recruitment and gene expression. Proteins are also able to interact with SUMOs non-covalently via their SUMO-interacting motifs [6, 58]. Through these interactions, SUMOylation can act as a reversible molecular “glue” in protein complex and biomolecular condensate formation [59, 60]. These mechanisms may also contribute to the control of cell identity.

Overall, our results not only deepen our understanding of SUMO biology but also suggest that targeted modulation of SUMOylation could enhance the efficiency and precision of cell fate engineering strategies.

## Supplementary Information

Below is the link to the electronic supplementary material.

Supplementary file1 (PDF 33190 KB)

Supplementary file2 SUMO2/3 chromatome members (XLSX 337 KB)

Supplementary file3 Chromatin-residing protein abundance analysis results (XLSX 1136 KB)

Supplementary file4 CEBPA chromatome members (XLSX 160 KB)

Supplementary file5 RNA-seq pairwise comparisons (XLSX 15760 KB)

Supplementary file6 RNA-seq Metascape pathway analysis results for pairwise comparisons (XLSX 126 KB)

Supplementary file7 RNA-seq Metascape pathway analysis results for PART modules (XLSX 267 KB)

## Data Availability

Raw and processed ChIP-seq, RNA-seq and ATAC-seq sequencing data have been deposited in NCBI’s Gene Expression Omnibus and are accessible through GEO Series accession numbers GSE311971, GSE311975 and GSE311969, respectively. The mass spectrometry proteomics data have been deposited to the ProteomeXchange Consortium via the PRIDE partner repository with the dataset identifiers PXD071373 (RIME) and PXD076495 (PAC).

## Abbreviations

BETA: Binding and expression target analysis
CEBPA: CCAAT/enhancer binding protein alpha
DEG: Differentially expressed gene
E2: β-Estradiol
ER: Estrogen receptor
FC: Fold change
iPSC: Induced pluripotent stem cell
MEF: Mouse embryonic fibroblast
PAC: Protein aggregation capture
PART: Partitioning algorithm based on recursive thresholding
PPI: Protein-protein interaction
PTM: Post-translational modification
RIME: Rapid immunoprecipitation mass spectrometry of endogenous proteins
SENP: SUMO-specific protease
SUMO: Small ubiquitin-like modifier
SUMOi: SUMOylation inhibitor ML-792
SPC: Spectral count
TF: Transcription factor

## Declarations

### Use of AI

Microsoft Copilot, an AI assistant built on OpenAI’s GPT-4 architecture, was utilized to enhance textual clarity and ensure grammatical accuracy. In analysis AI was utilized to recategorize pathways and to assist in the implementation of R scripts.
