# Supplementary material for: Regulation of lineage reprogramming by dynamic chromatin SUMOylation
Source: Cell Mol Life Sci. 2026 May 27;83(1):289. doi: 10.1007/s00018-026-06255-5 (PMC13396097; doi:10.1007/s00018-026-06255-5)
Supplement: Supplementary file 1 — Supplementary file1 (PDF 33190 KB) [file 18_2026_6255_MOESM1_ESM.pdf]

Supplementary figures

for

Regulation of lineage reprogramming by dynamic chromatin SUMOylation

Emma Valima, A.B.M. Kaiser Manjur, Eevi Savinainen, Vera Varis, Kaisa-Mari Launonen, Thomas Graf, Markku Varjosalo, Einari A. Niskanen and Jorma J. Palvimo

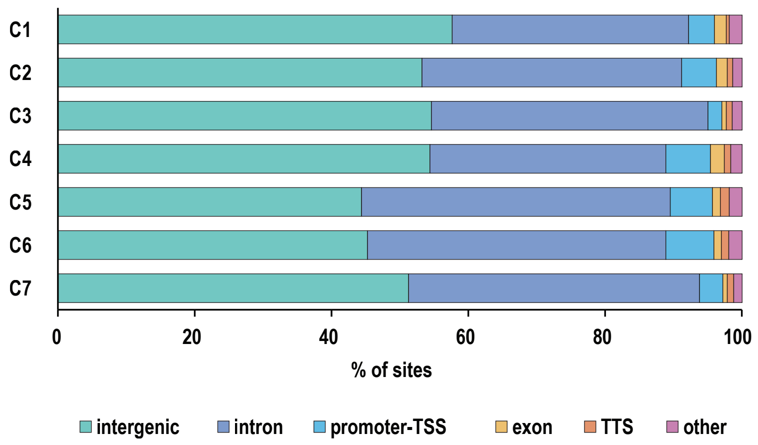

Supplementary Figure S1. Genomic distribution of SUMO2/3 binding sites in C1-7.

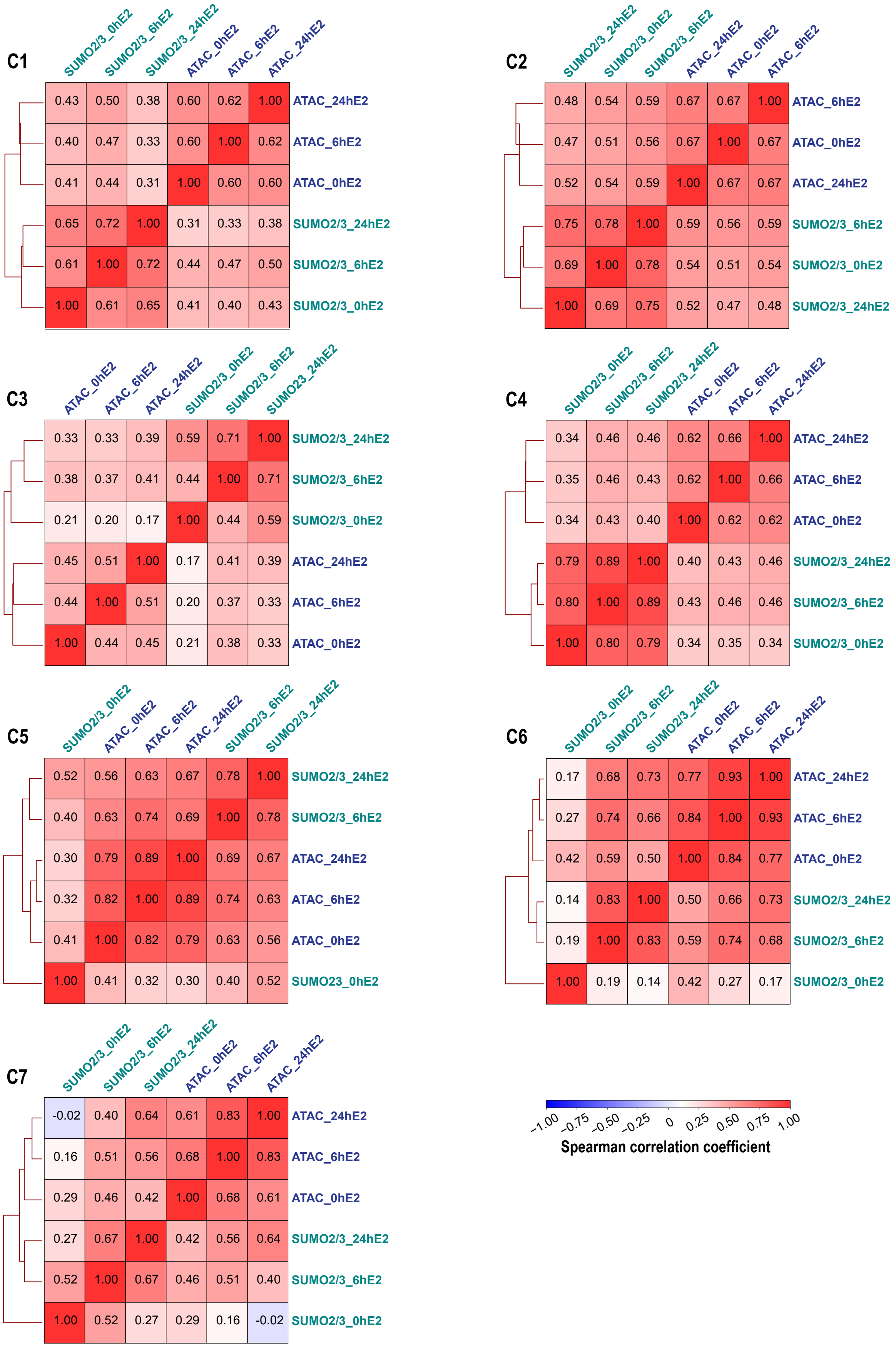

Supplementary Figure S2. Correlation analyses between SUMO2/3 ChIP-seq and ATAC-seq read coverages in DMSO in SUMO2/3 binding clusters C1-7. Hierarchically clustered heatmaps display Spearman correlations between all pairwise comparisons. Number and colour in each cell indicates the Spearman correlation coefficient.

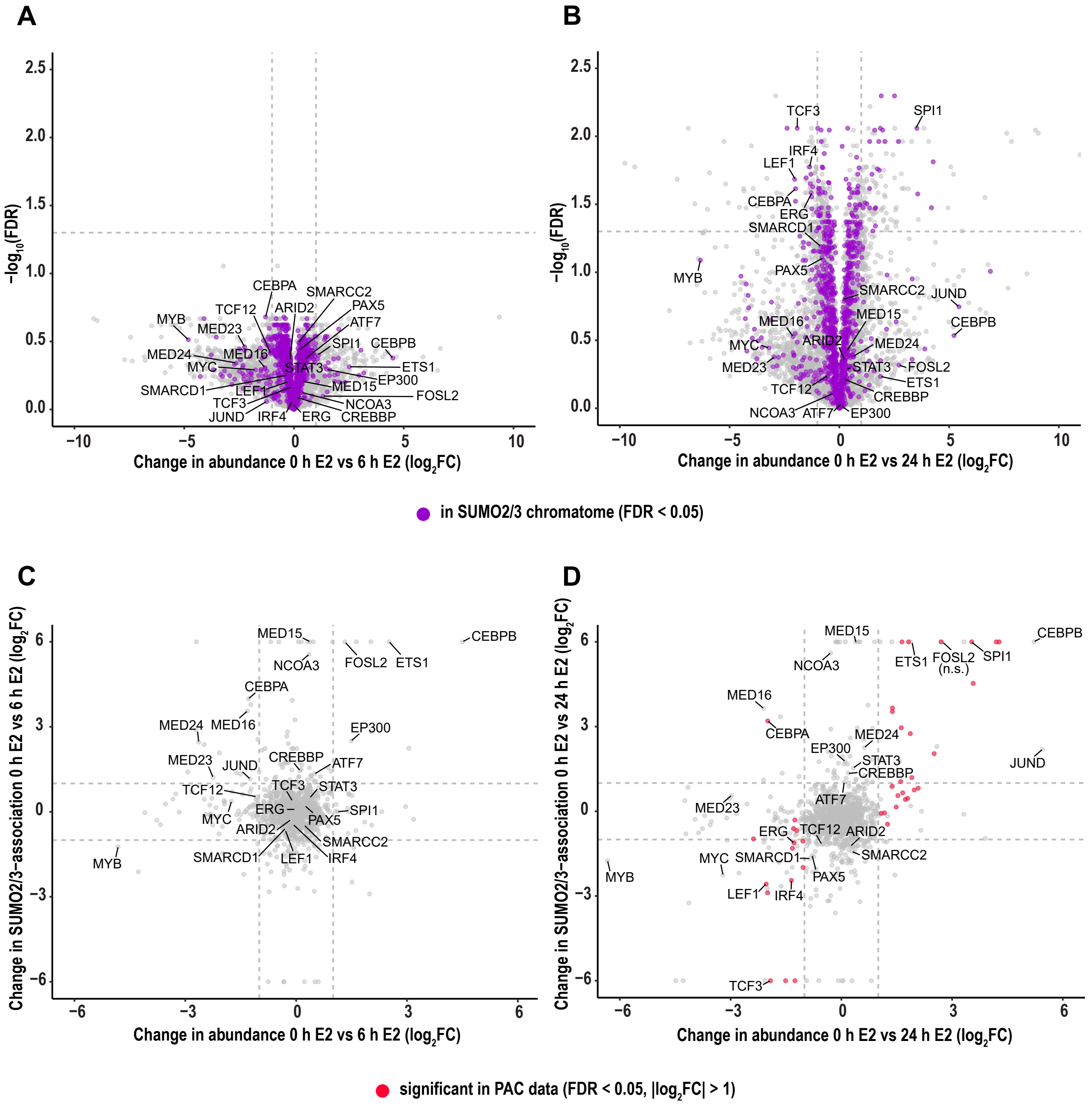

Supplementary Figure S3. Comparisons of chromatin-residing protein abundace data obtained using protein-aggregation capture (PAC) and SUMO2/3 chromatome data obtained using RIME. Changes in protein abundance at 6 h (A) and 24 h (B) after induction of transdifferentiation. Volcano plots depict all proteins detected with PAC (n=5 418), with proteins found also in the SUMO2/3 chromatome (FDR < 0.05) denoted in purple. X-axis represents log₂FC in protein abundance at the indicated timepoints and y-axis represents $-\log_{10}$(FDR). Horizontal dashed line represents $-\log_{10}$ transformation of signficance threshold (FDR < 0.05) in PAC data. Selected SUMO2/3 chromatome members are annotated. (C) and (D) Scatterplots of proteins present in both PAC and SUMO2/3 chromatome data (n=1 099). X-axis represents $\log_2$FC in protein abundance (PAC) and y-axis represents $\log_2$FC in SUMO2/3 association (RIME data) at 6 h (C) and 24 h (D) after induction of transdifferentiation. Proteins with significant changes in abundance (FDR < 0.05, |log2FC| > 1) are denoted in red. Selected SUMO2/3 chromatome members are annotated. n.s. = not significant (overlapping data points)

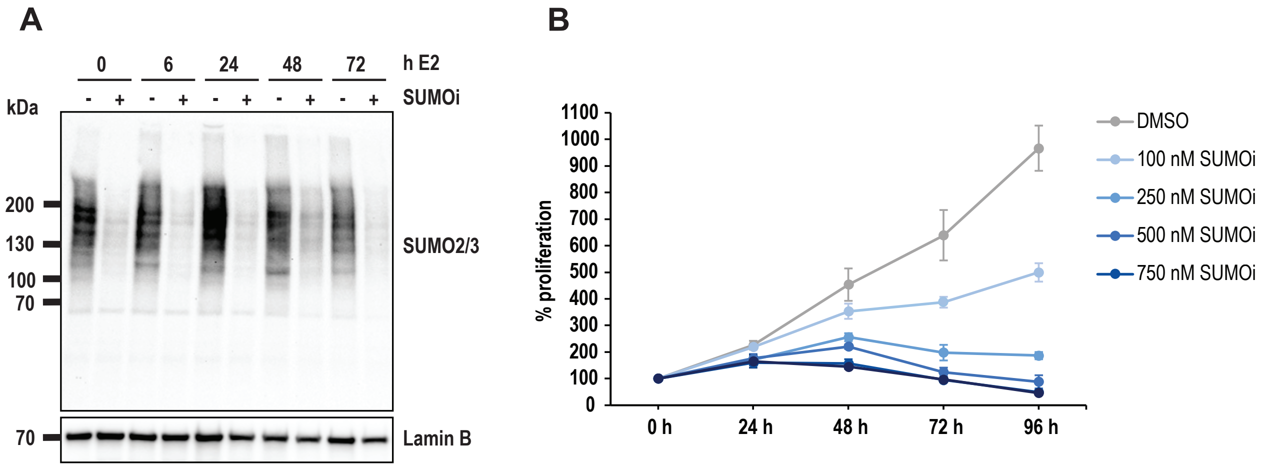

Supplementary Figure S4. (A) SUMO2/3 immunoblot of E2-induced BLaER1 cells at indicated timepoints with or without SUMOi (100 nM). (B) MTS assay of uninduced BLaER1 cells exposed to indicated concentrations of SUMOi.

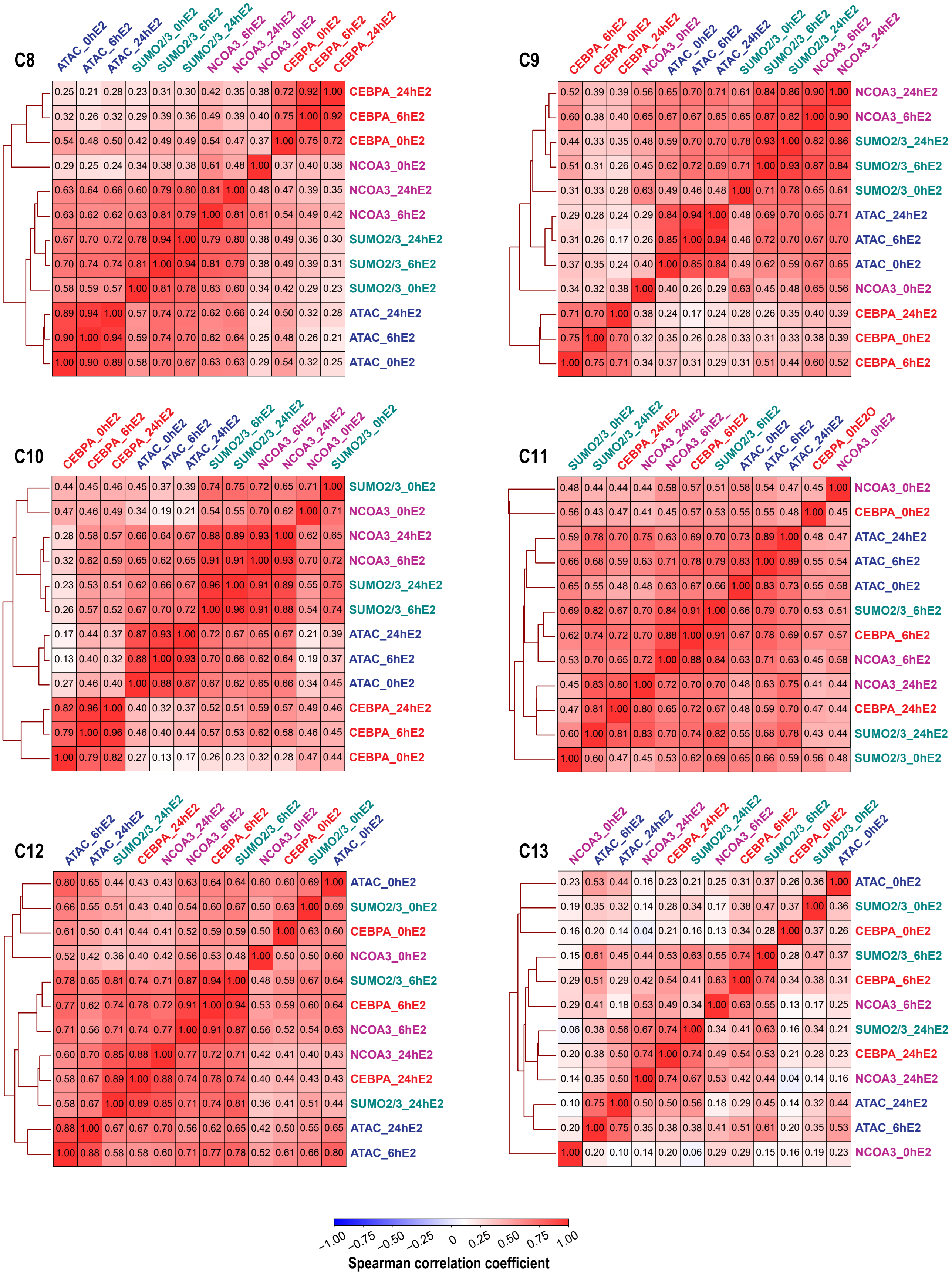

Supplementary Figure S5. Correlation analyses between ChIP-seq and ATAC-seq read coverages in DMSO in CEBPA binding clusters C8-13. Hierarchically clustered heatmaps display Spearman correlations between all pairwise comparisons. Number and colour in each cell indicates the Spearman correlation coefficient.

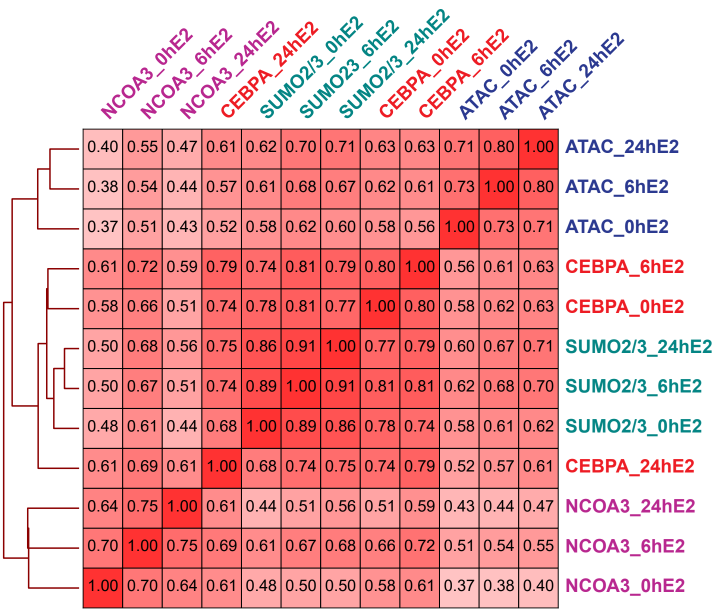

Supplementary Figure S6. Genome-wide correlation analyses between ChIP-seq and ATAC-seq read coverages in DMSO. Hierarchically clustered heatmaps display Spearman correlations between all pairwise comparisons. Number and colour in each cell indicates the Spearman correlation coefficient. Spearman correlations were calculated in 10 kb bins across the genome.

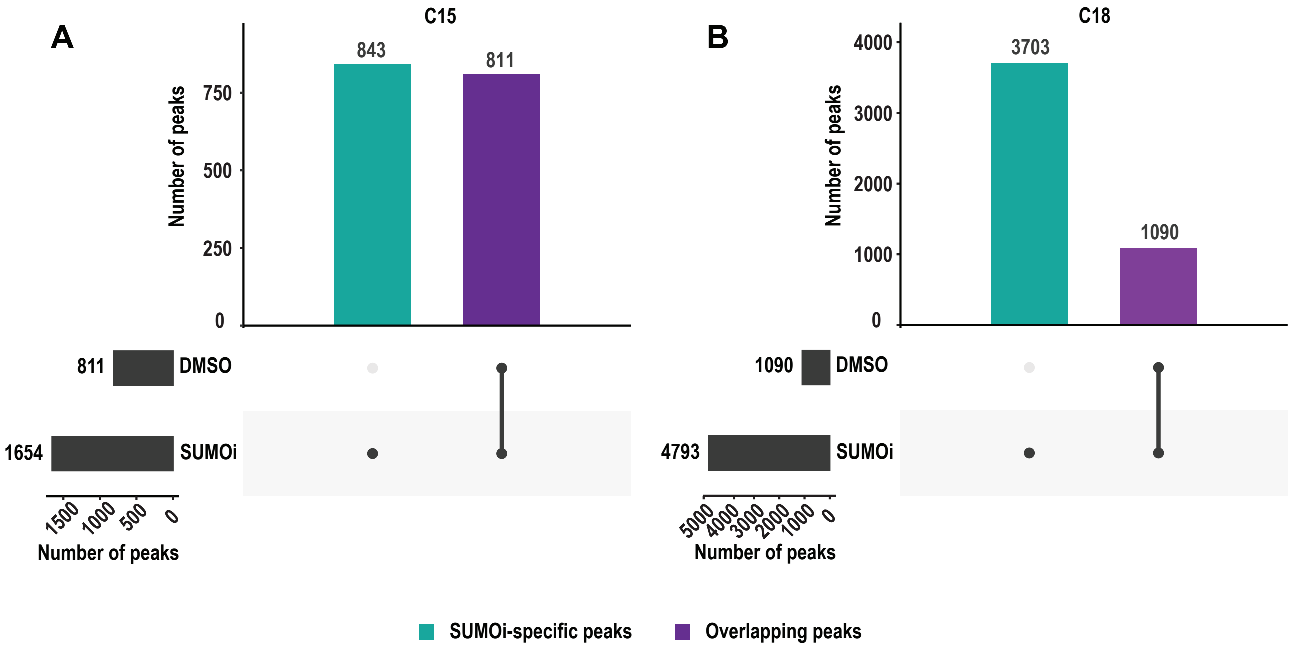

Supplementary Figure S7. Upset plots presenting the number of SUMOi-specific (detected only in SUMOi) and overlapping peaks (detected both in DMSO and SUMOi) in CEBPA binding clusters C15 (A) and C18 (B).

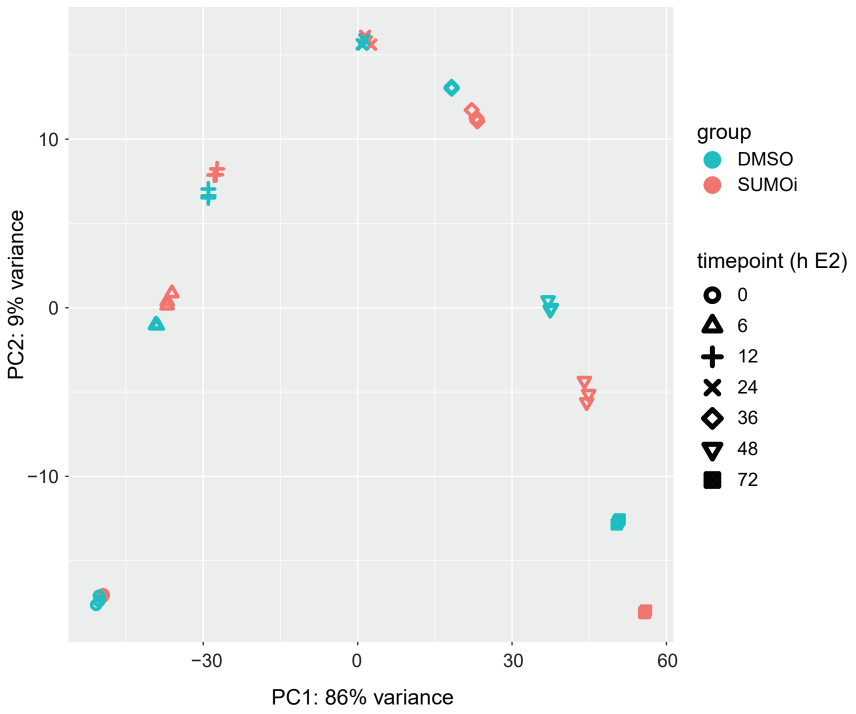

Supplementary Figure S8. Principal component analysis (PCA) of BLaER1 RNA-seq data.

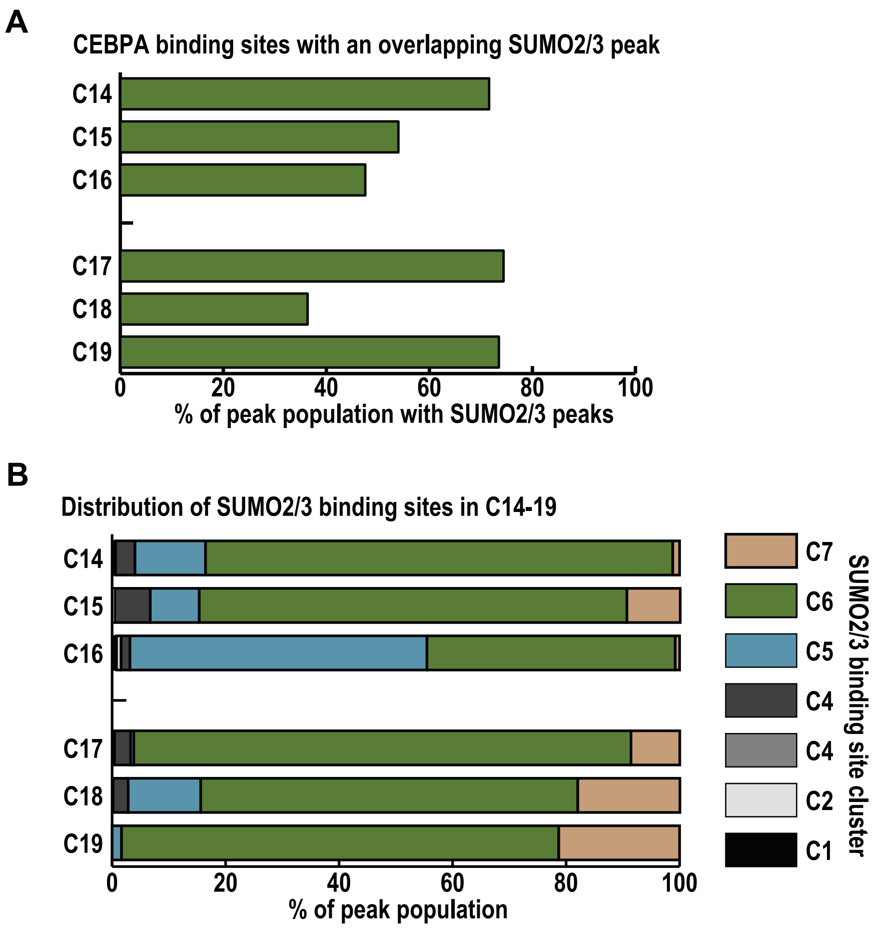

Supplementary Figure S9. (A) Percentages of CEBPA binding sites with an overlapping SUMO2/3 peak in C14-19. (B) Distribution of overlapping SUMO2/3 and CEBPA binding sites in C14-19 vs. C1-7.

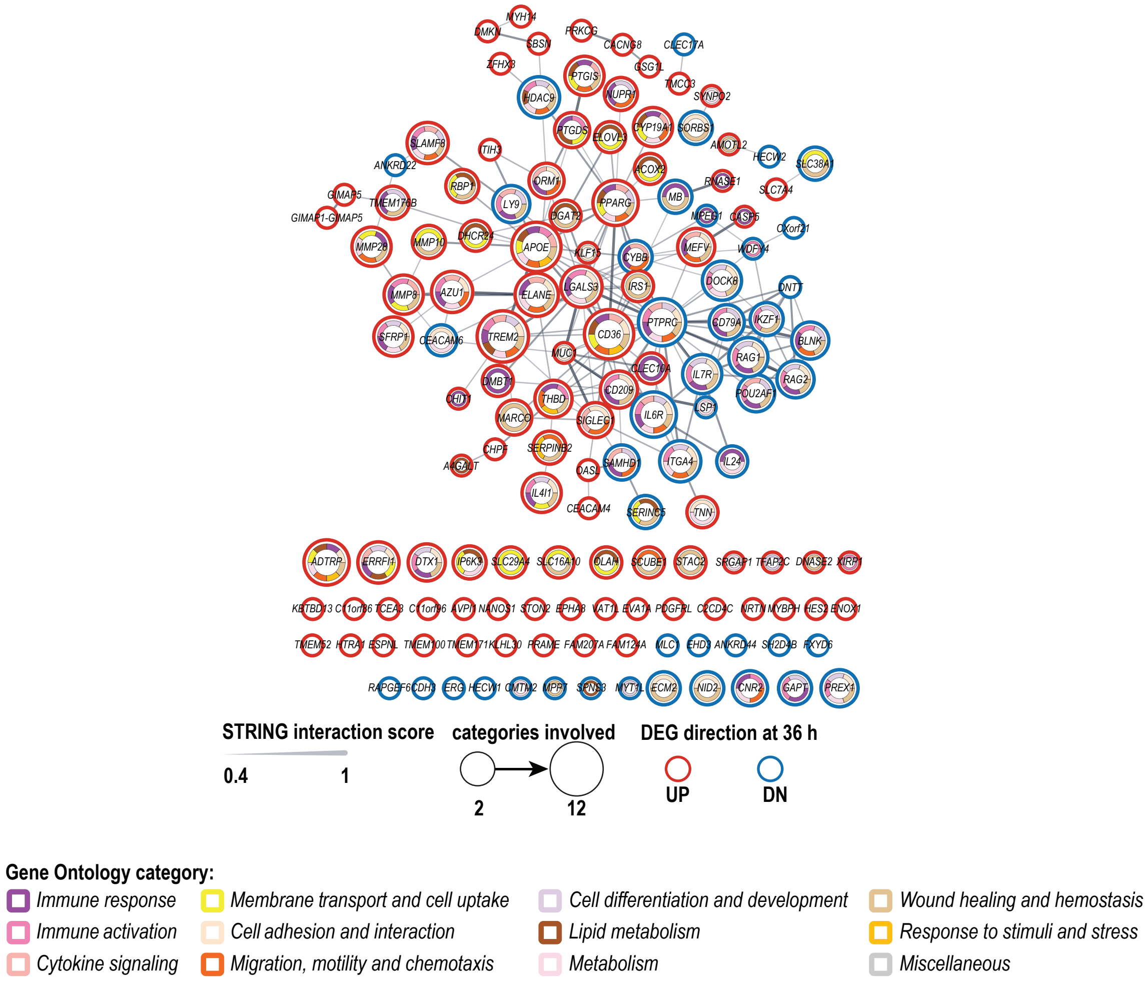

Supplementary Figure S10. Protein-protein interactions of all proteins coded by DEGs associated with overlapping CEBPA and SUMO2/3 binding sites. Pie plots indicated which Gene Ontology categories each protein is enriched for; if empty, protein was not enriched for any of the listed categories. Interactions were retrieved from STRING database.

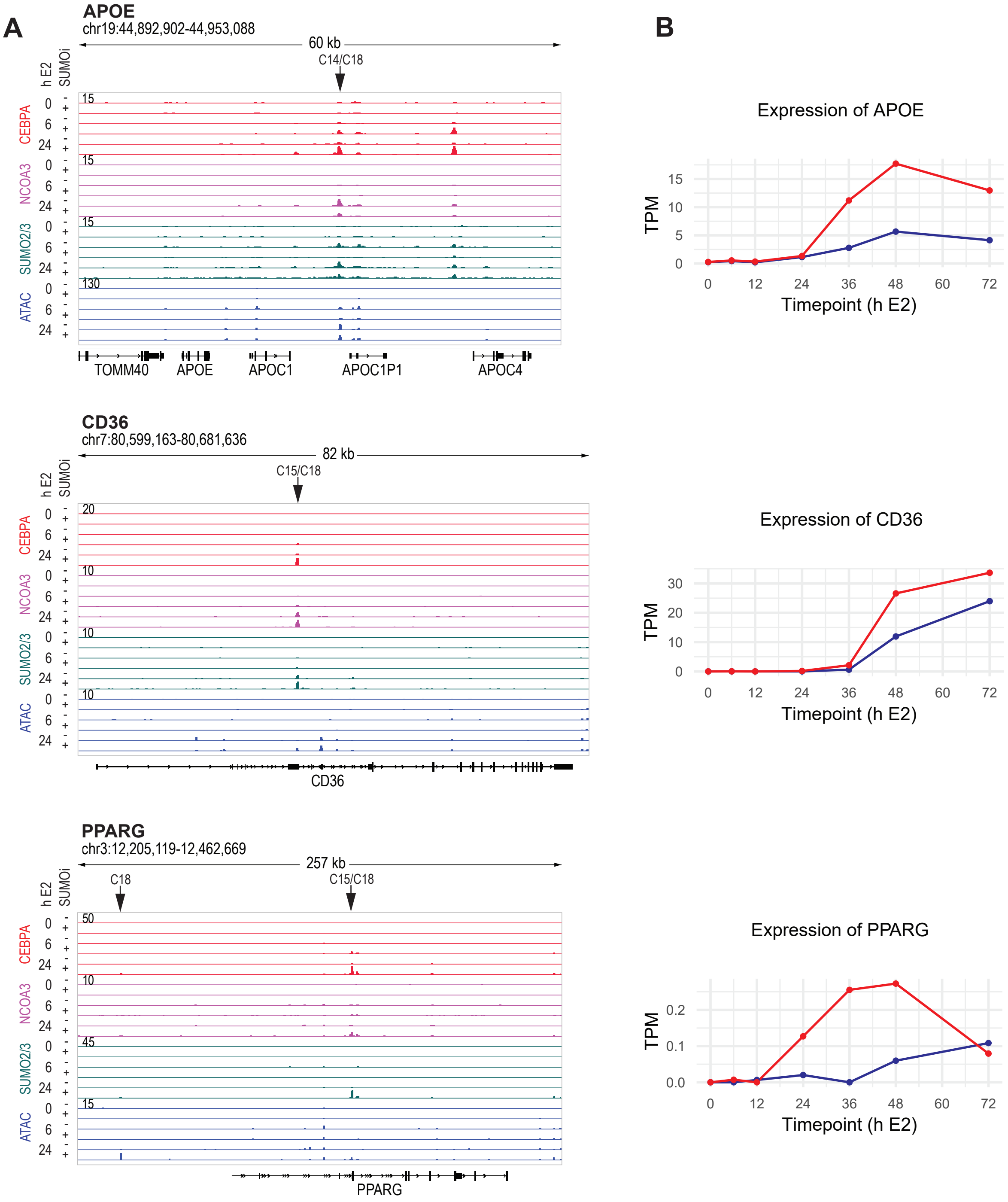

Supplementary Figure S11. (A) IGV tracks of CEBPA, NCOA3 and SUMO2/3 ChIP-seq signal and ATAC-seq signal on APOE, CD36 and PPARG loci. Arrows denote CEBPA peaks from indicated binding site clusters with overlapping SUMO2/3 peak. (B) Expression trajectories of APOE, CD36 and PPARG.

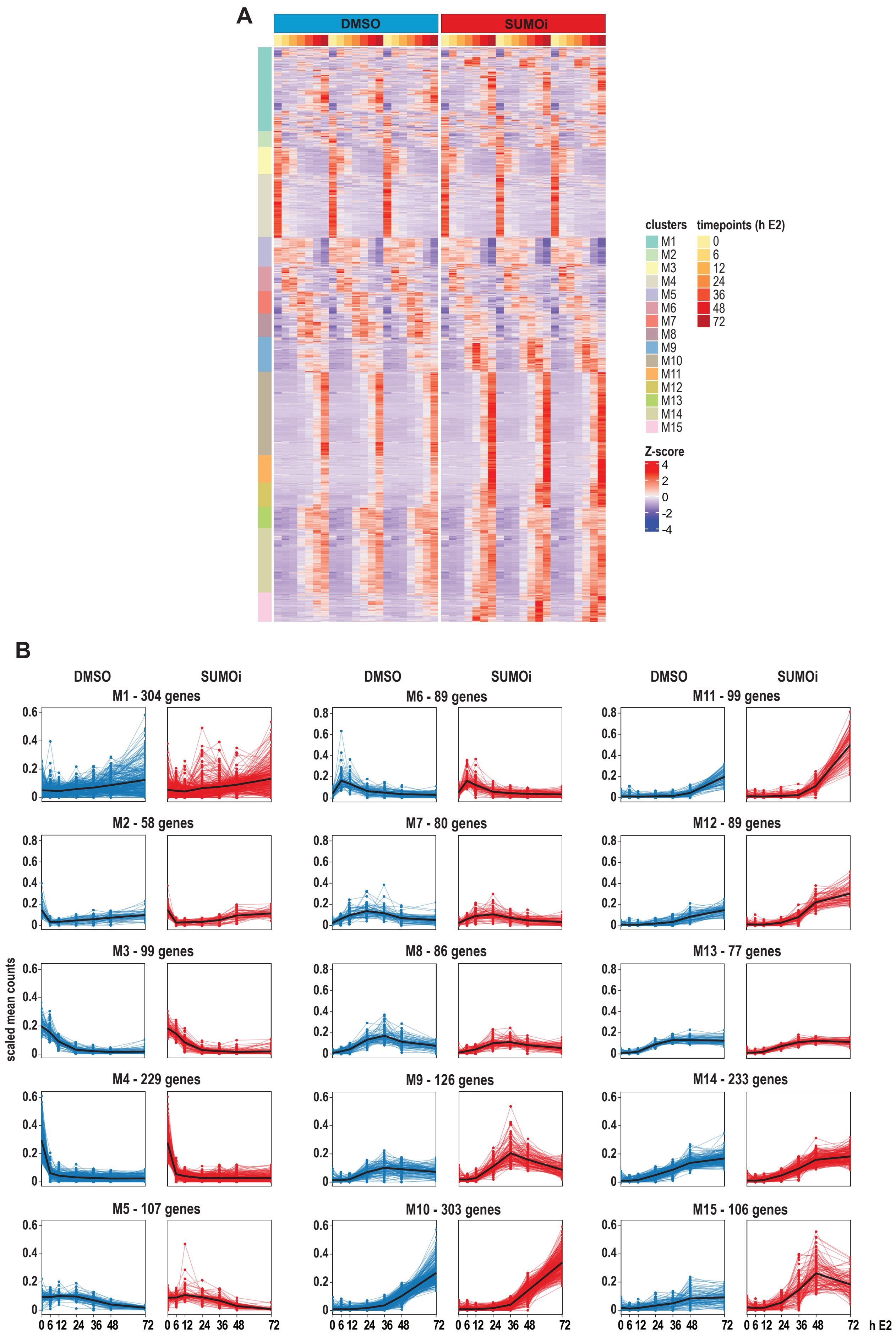

Supplementary Figure S12. (A) Heatmap of co-expressed gene modules from PART analysis of BLaER1 RNA-seq data. (B) Expression trajectories for modules M1-15.

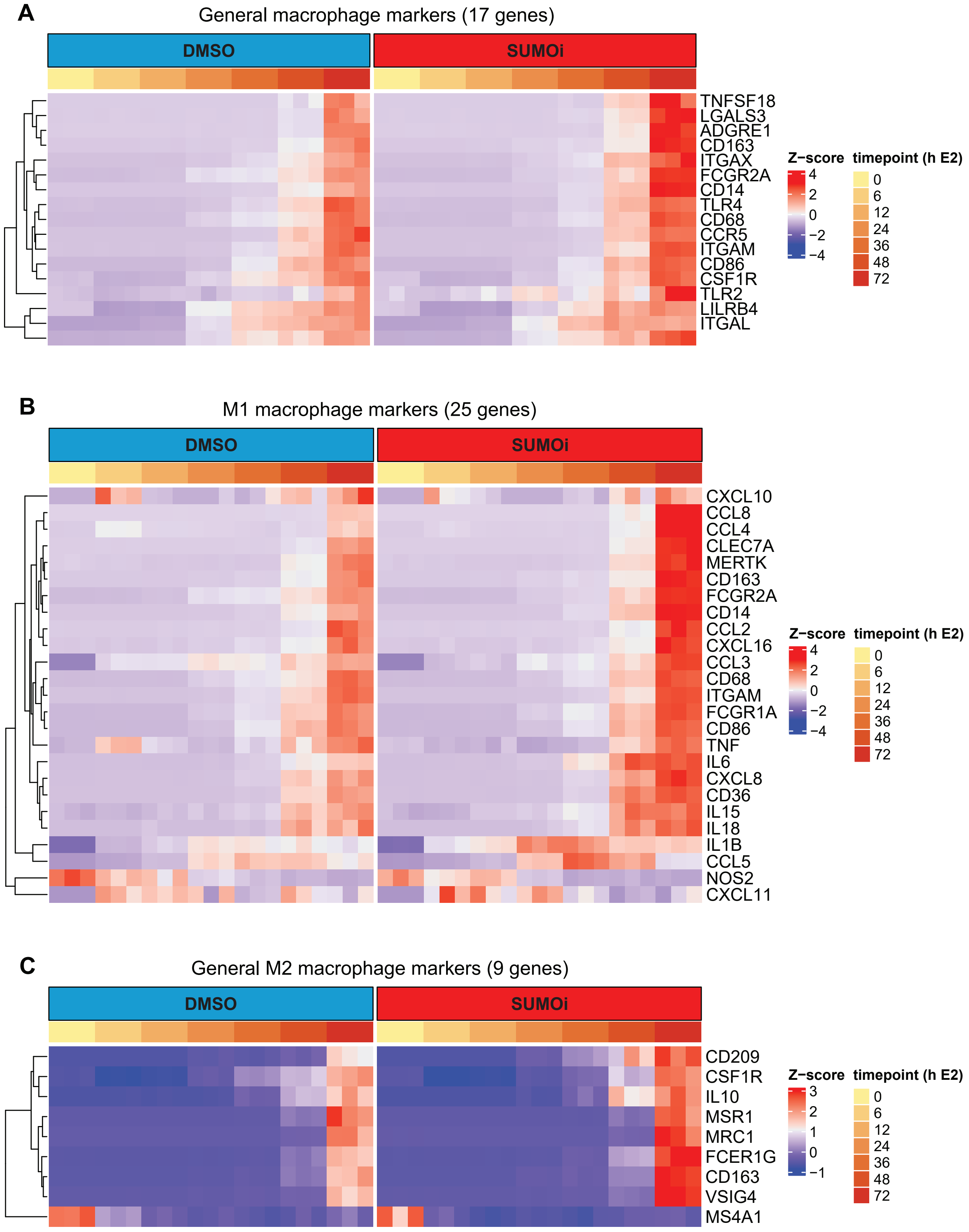

Supplementary Figure S13. Heatmaps of differentially expressed ($|\log_2 FC| > 1.5$, adj. p-value < 0.01) (A) general macrophage marker genes, (B) M1 type macrophage marker genes, and (C) general M2 type macrophage marker genes in BLaER1 cells.
